# Selective Recruitment of Nuclear Factors to Productively Replicating Herpes Simplex Virus Genomes

**DOI:** 10.1371/journal.ppat.1004939

**Published:** 2015-05-27

**Authors:** Jill A. Dembowski, Neal A. DeLuca

**Affiliations:** Department of Microbiology and Molecular Genetics, University of Pittsburgh School of Medicine, Pittsburgh, Pennsylvania, United States of America; University of Glasgow, UNITED KINGDOM

## Abstract

Much of the HSV-1 life cycle is carried out in the cell nucleus, including the expression, replication, repair, and packaging of viral genomes. Viral proteins, as well as cellular factors, play essential roles in these processes. Isolation of proteins on nascent DNA (iPOND) was developed to label and purify cellular replication forks. We adapted aspects of this method to label viral genomes to both image, and purify replicating HSV-1 genomes for the identification of associated proteins. Many viral and cellular factors were enriched on viral genomes, including factors that mediate DNA replication, repair, chromatin remodeling, transcription, and RNA processing. As infection proceeded, packaging and structural components were enriched to a greater extent. Among the more abundant proteins that copurified with genomes were the viral transcription factor ICP4 and the replication protein ICP8. Furthermore, all seven viral replication proteins were enriched on viral genomes, along with cellular PCNA and topoisomerases, while other cellular replication proteins were not detected. The chromatin-remodeling complexes present on viral genomes included the INO80, SWI/SNF, NURD, and FACT complexes, which may prevent chromatinization of the genome. Consistent with this conclusion, histones were not readily recovered with purified viral genomes, and imaging studies revealed an underrepresentation of histones on viral genomes. RNA polymerase II, the mediator complex, TFIID, TFIIH, and several other transcriptional activators and repressors were also affinity purified with viral DNA. The presence of INO80, NURD, SWI/SNF, mediator, TFIID, and TFIIH components is consistent with previous studies in which these complexes copurified with ICP4. Therefore, ICP4 is likely involved in the recruitment of these key cellular chromatin remodeling and transcription factors to viral genomes. Taken together, iPOND is a valuable method for the study of viral genome dynamics during infection and provides a comprehensive view of how HSV-1 selectively utilizes cellular resources.

## Author Summary

HSV-1 is a human pathogen that infects over 50% of the population. The virus persists as a latent infection in the ganglia of an infected host and upon stressful conditions is reactivated to a lytic state in which it causes recurrent sores at the initial site of infection. During lytic infection, HSV highjacks the host cell to propagate its genome and produce new virus particles. However, there is limited knowledge of what cellular proteins interact with and function on the viral genome. We therefore developed methods to purify viral genomes from productively infected cells to identify associated viral and cellular proteins. We found proteins and protein complexes that have previously been implicated in HSV infection to be enriched on viral genomes, as well as several novel proteins that are likely involved in productive infection. These data provide valuable insight into HSV biology. Furthermore, these methods can be adapted to study other viruses, as well as other aspects of the HSV life cycle.

## Introduction

The genomes of eukaryotic DNA viruses vary in complexity with respect to the number of genes they encode, and hence their dependence on host-cell functions. With the exception of poxviruses, all replicate in the cell nucleus and therefore utilize the nuclear machinery for the maintenance, replication, and expression of their genomes. The dynamic interactions between viral and cellular proteins and the viral genome, function to mediate the different steps in the life cycle of the virus, and hence determine the outcome of infection. These include interactions that mediate the entry of the genome into the nucleus, its expression and replication, and ultimately the packaging of nascent genomes in capsids.

Herpes simplex virus 1 (HSV-1) has a linear genome comprised of 152 kilobasepairs [1,2]. It enters the nucleus from the capsid through pores in the nuclear envelope [3–5]. The genome then participates in a series of interactions that results in a nucleo-protein complex near ND10 structures [6]. Here, the genome is susceptible to activities of the intrinsic cellular antiviral response. The genome also contains nicks and gaps, and these along with the genomic termini elicit a DNA damage response, the nature of which may be consequential to viral infection [7]. Viral genomes initially associate with ND10 structures, where through the action of ICP0, ND10 proteins are degraded or dispersed resulting in the prerequisite structure for efficient transcription and replication [6,8]. Viral DNA replication then results in the formation large replication compartments, which fill the host nucleus and concentrate viral and cellular factors to replicating viral genomes [9].

HSV-1 encodes two transcription factors, VP16 [10,11] and ICP4 [12], which function along with the cellular RNA polymerase II transcription machinery [13] to transcribe the viral genome. These factors initially colocalize with prereplicative genomes [14–16] and these interactions as well as those involving viral and cellular RNA-processing factors result in an ordered cascade of viral gene expression [17,18]. Seven HSV gene products are sufficient in cells to replicate DNA in an HSV-origin dependent manner [19]. While this set of viral proteins includes a DNA-dependent DNA polymerase and other functional analogs of cellular DNA replication proteins, it is not sufficient to drive origin-dependent replication *in vitro*, suggesting the requirement for as yet unknown cellular proteins [20]. Finally, an additional set of proteins interacts with the genome in the processes of cleaving unit length genomes and their packaging in capsids [21]. These processes have been, and continue to be the focus of studies in many laboratories since significant gaps exist in our understanding of all these processes, and how they ultimately contribute to viral multiplication and pathogenesis. A shortcoming contributing to these gaps is our relative lack of knowledge of the proteins, particularly cell-derived, which interact with viral genomes in different phases of infection.

Recently, ethynyl-modified nucleosides along with click chemistry and immunofluorescence were used to trace the fate of input adenovirus genomes in infected cells [22]. Nucleoside analogs were also incorporated into replicating herpes simplex and vaccinia viral DNA to demonstrate that this technique can be used to label other viral genomes and could potentially be used to track these genomes throughout infection. In addition, ethynyl-modified nucleosides have been used in a procedure known as isolation of proteins on nascent DNA (iPOND) to identify the proteins at cellular replication forks [23–28]. This procedure involves the metabolic incorporation of 5-ethynyl-2´-deoxyuridine (EdU) into the DNA, biotinylating the EdU-labeled DNA by click chemistry, followed by the affinity purification of the biotinylated DNA, and the subsequent analysis of the proteins associated with it. We have adopted and modified these procedures to enable the visualization of the HSV genome at different stages of infection, as well as the interrogation of the viral and cellular proteins on replicated/replicating viral genomes. The results elucidate the viral and cellular proteins associating with viral DNA during infection and provide a comprehensive view of the cellular machinery functioning on HSV genomes.

## Results

### Labeling and imaging HSV genomes

Ethynyl-modified nucleosides have been used to prelabel and then track single incoming adenovirus genomes within infected cells [22]. While this approach was also used to examine HSV genomes in replication compartments, input genomes were not imaged. We sought to determine if ethynyl-modified nucleosides could be used to label and track HSV genomes during early (before DNA replication), as well as late (after DNA replication) stages of infection. We also intended to use viral DNA imaging to optimize HSV genome labeling for purification of viral genomes by iPOND.

Preliminary experiments demonstrated that EdU and EdC were poorly incorporated in HSV DNA. We hypothesized that deletion of the HSV deoxyuridine triphosphatase (dUTPase) and uracil glycosylase enzymes would increase incorporation into the viral genome. HSV-1 uracil glycosylase and dUTPase mutant strains were generated by introducing premature termination codons early in the reading frames of the UL2 and UL50 genes (Fig A in S1 Text). As found for labeling of adenovirus genomes [22], ethynyl nucleoside incorporation into HSV genomes resulted in slightly reduced virus titers (Fig B in S1 Text). The same concentrations of EdU or EdC had a greater effect on the titer of the UL2/UL50 double mutant virus than on wild type KOS, suggesting that the double mutant is more efficiently labeled by both EdU and EdC. EdU was used in all subsequent experiments.

**Fig 1:**
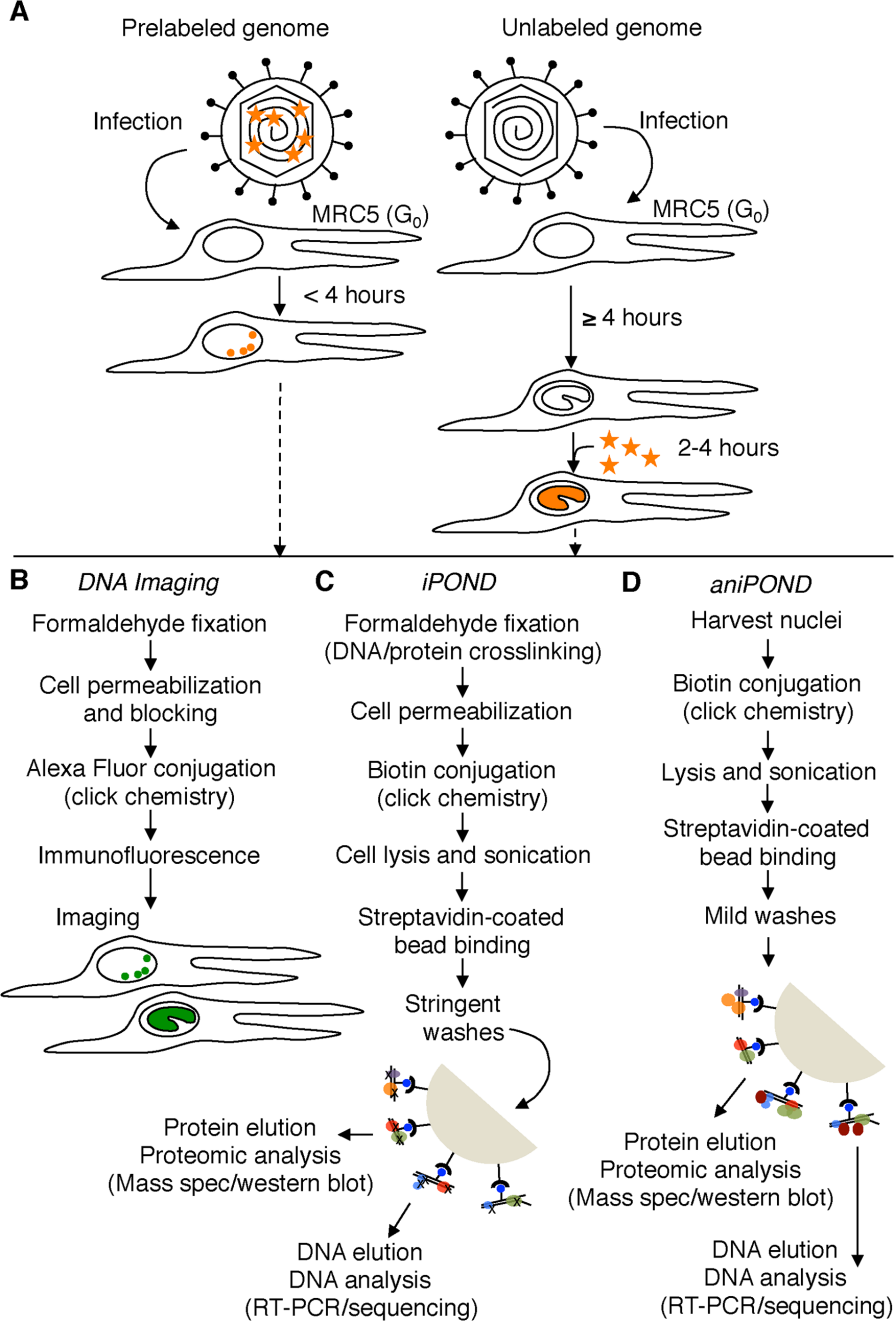
Schematic representation of procedures used in this paper. (A) Resting MRC-5 cells in G_0_ were infected with either prelabeled (left) or unlabeled (right) virus. To assay unreplicated viral DNA (left), prelabeled genomes were processed less than four hpi. To assay viral replication compartments (right), EdU (orange stars) was added to the growth medium during viral DNA replication (≥ 4 hpi) and genomes were assayed 2–4 hours after the addition of EdU. EdU labeled DNA is orange. (B) Viral and cellular DNA, as well as viral and cellular proteins were labeled and visualized as described in the experimental procedures. DNA imaging experiments were carried out in proliferating Vero cells. Viral DNA is green. (C) and (D) iPOND and aniPOND experiments were carried out as described. aniPOND (accelerated native iPOND) is a modified version of iPOND that does not involve crosslinking and therefore requires less stringent wash conditions during purification.

**Fig 2:**
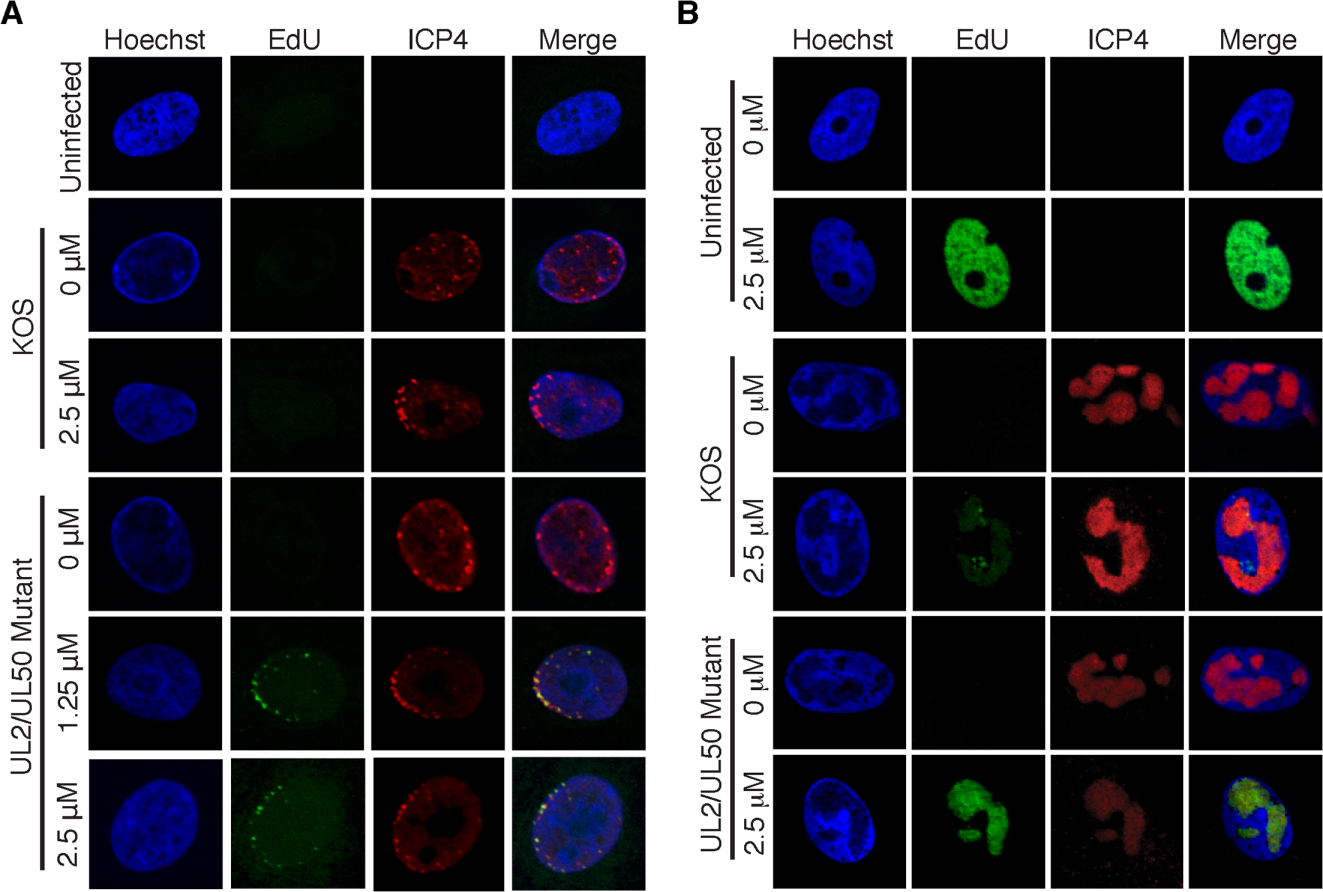
Visualization of EdU labeled HSV-1 genomes. (A) Prelabeled input viral genomes were visualized in the nucleus of infected cells 2 hpi. Vero cells were infected with wild type KOS or the UL2/UL50 mutant virus carrying unlabeled (0 μM) or prelabeled viral genomes. Labeled virus stocks were generated by growing KOS or UL2/UL50 mutant in the presence of 1.25 or 2.5 μM EdU as described in the experimental procedures. Cellular DNA was visualized by Hoechst staining, viral DNA by click chemistry with EdU, and ICP4 by immunofluorescence. Merged panels show colocalization of viral DNA with ICP4. Prelabeled KOS DNA could not be detected under these conditions. (B) Cells infected with KOS or UL2/UL50 mutant were grown in the presence of 0 or 2.5 μM EdU for 4–8 hpi. Uninfected cells were grown in the presence of EdU for 4 hours. DNA imaging was as described in (A).

To compare the relative amount of EdU incorporated into wild type and mutant genomes, we carried out viral infection and DNA imaging as outlined in Fig 1A and 1B. EdU labeled input genomes and replication compartments were tagged with Alexa Fluor 488 by click chemistry and visualized by fluorescence microscopy (Fig 2). Prelabeled UL2/UL50 mutant genomes that colocalized with the viral transcription factor, ICP4, were visualized in the nucleus of infected cells (Fig 2A). The distribution of ICP4 foci two hours after infection largely resembles that observed previously after infection with HSV-1 at a MOI of 10 PFU/cell [29]. Using these same conditions, we were unable to detect KOS genomes prelabeled with 2.5 μM EdU (Fig 2A). We also visualized viral replication compartments that colocalize with ICP4 8 hpi in both wild type KOS and UL2/UL50 mutant virus infected cells (Fig 2B). While it was possible to detect EdU labeling coinciding with ICP4 staining in KOS infected cells, significantly more was observed with the UL2/UL50 mutant. Taken together, the UL2/UL50 mutant virus incorporates more EdU into its genome during DNA replication and allows for more sensitive imaging of HSV-1 viral DNA during infection.

### Optimization and validation of HSV iPOND

To identify the viral and cellular proteins that function on viral DNA at different stages of infection, we adapted the iPOND method [25] for analysis of viral genomes and associated proteins. To optimize viral iPOND, we initially considered several factors. Proliferating cells grown in the presence of EdU incorporate EdU into their genomes during DNA replication (Fig 2B, uninfected, 2.5 μM). Therefore, conditions in which viral DNA, but not cellular DNA is labeled in the presence of EdU were established. Addition of EdU to the growth medium of proliferating Vero cells that were mock- or HSV-1-infected resulted in labeling of 65% or 29% of cellular genomes (Fig C in S1 Text, panels Vero cells), respectively. HSV infection inhibits G1/S and G2/M phases of the cell cycle [30,31] consistent with less labeling of cellular DNA in infected cells. In contrast to proliferating cells, less than 1% of cellular genomes were labeled with EdU when human MRC-5 fibroblast cells that were grown to confluency were infected with HSV in the presence of EdU (Fig C in S1 Text, panels MRC-5 cells). Therefore, resting MRC-5 cells were used in iPOND experiments to avoid contamination with cellular DNA. These cells also have the added advantage that they are a natural host to lytic HSV infection and they do not express high levels of cellular glycosylases to limit cleavage of labeled viral genomes [32].

One of the limitations of iPOND is that a large amount of EdU-labeled DNA is required to pull down enough protein for proteomic analysis [25]. Because the UL2/UL50 mutant virus is more efficiently labeled with EdU than wild type virus, we hypothesized that more viral DNA and associated proteins could be purified by iPOND of the mutant virus. We tested iPOND for the purification of proteins associated with genomes of wild type KOS, UL2 and UL50 single mutant, and UL2/UL50 double mutant viruses (outlined in Fig 1C). The relative protein yield for each virus was compared by western blot for the viral transcription factor ICP4 (Fig D in S1 Text). ICP4 associates with viral genomes throughout infection and is a good indicator of protein yield. The negative control was iPOND carried out on virus-infected cells incubated in the absence of EdU. For all viruses tested, ICP4 was not detected in the negative control, but was detected when iPOND was carried out on viral genomes that were labeled with EdU. The greatest relative amount of ICP4 was detected with the UL2/UL50 mutant virus, consistent with fluorescence imaging of labeled viral genomes (Fig 2B). Therefore, the UL2/UL50 mutant virus was used for iPOND experiments.

**Fig 3:**
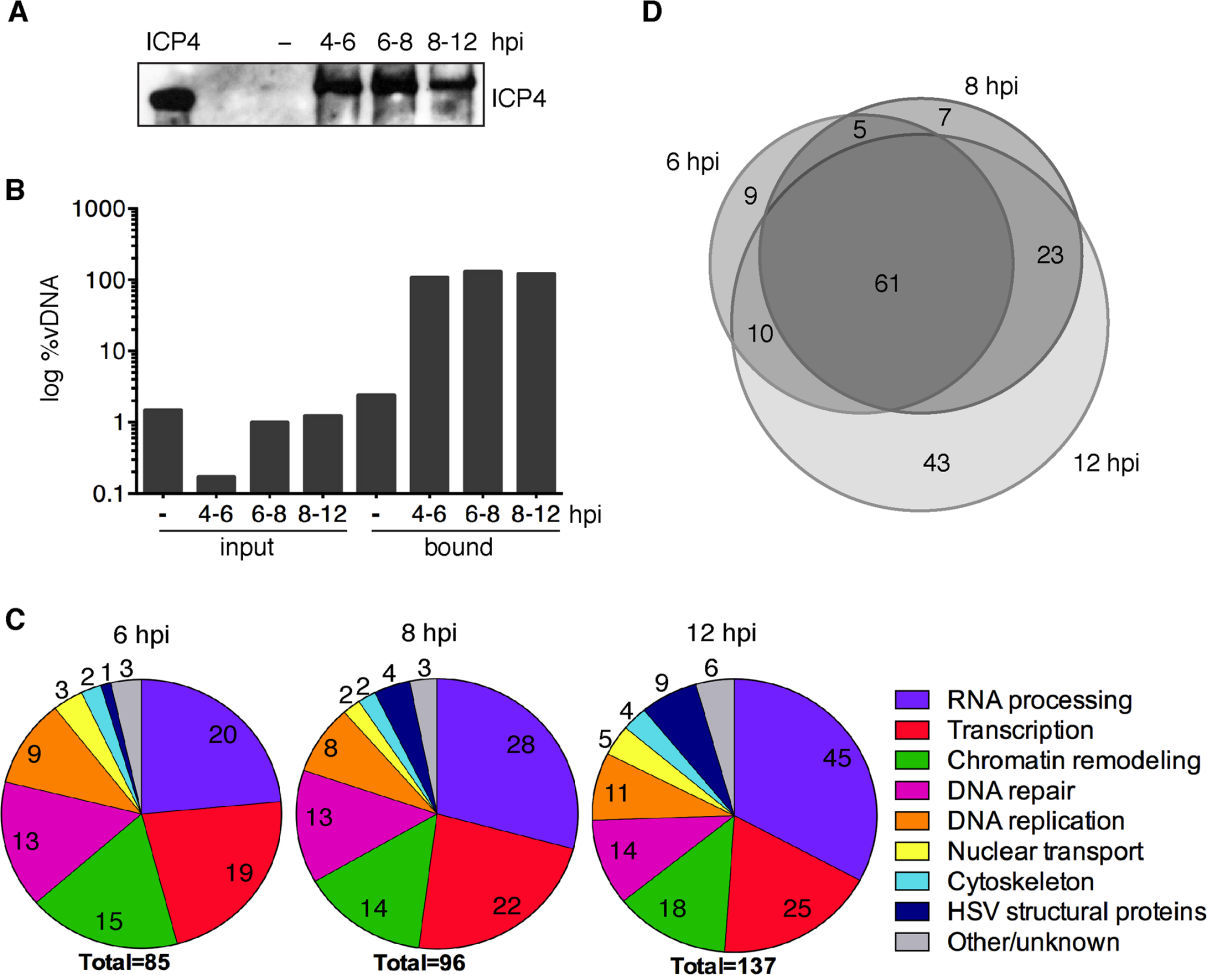
iPOND detects viral and cellular proteins associated with replicated HSV-1 genomes. (A) ICP4 was detected by western blot of protein eluates from iPOND carried out on viral genomes grown in the presence of EdU at 4–6, 6–8, and 8–12 hpi. The control was iPOND carried out on virus grown in the absence of EdU (−) and harvested 8 hpi. Purified ICP4 is shown. (B) DNA eluted from streptavidin-coated beads during iPOND experiments in (A) is viral. The amount of viral DNA present in cell lysates (input) and eluted from beads (bound) during iPOND experiments was measured by qRT-PCR of the viral thymidine kinase (TK) gene. The ratio of viral DNA (vDNA) to total DNA was calculated and is presented as log %vDNA. (C) Pie charts summarize proteins that were identified by mass spectrometry of protein eluates from iPOND carried out 6, 8, and 12 hpi with UL2/UL50 mutant virus. Values indicate the number of proteins identified for each functional category. (D) Venn diagrams depict the overlap of proteins identified by iPOND at each time point.

To identify the proteins associated with viral genomes by iPOND, we labeled viral DNA at three time points during DNA replication. EdU was added to the medium of infected cells at 4–6, 6–8, or 8–12 hpi and cells were fixed for iPOND at 6, 8, or 12 hpi, respectively. Proteins recovered by iPOND were probed for ICP4 by western blotting (Fig 3A). ICP4 was detected at all time points, but not in the unlabeled negative control. To ensure that DNA isolated by iPOND was viral, input DNA from cell lysates and DNA bound to streptavidin-coated beads was extracted, the amount of viral DNA was measured, and the percentage of viral/total DNA was calculated (Fig 3B). DNA eluted from beads during iPOND experiments was nearly 100% viral in nature. This is a significant enrichment compared to input DNA (0.2–1.5% viral). To determine if the entire viral genome was labeled and purified in our assays, high throughput DNA sequencing was carried out on DNA eluted from streptavidin-coated beads (Fig E in S1 Text). At all time points, the distribution of bead-bound DNA was relatively homogeneous across the viral genome. Taken together, iPOND should enable the specific purification of proteins associated with the entire replicated HSV-1 genome.

### Factors that mediate host cell nuclear processes are enriched on HSV-1 genomes during viral DNA replication

To determine the identity of proteins bound to viral genomes at 6, 8, and 12 hpi, mass spectrometry was carried out on proteins that were crosslinked to viral DNA and purified by iPOND. Two independent iPOND experiments were carried out for each time point, each with an unlabeled virus negative control that was prepared on the same day with the same cells, virus, and reagents. Proteins were considered significantly enriched on viral genomes if they were identified with high confidence in duplicate experiments to be enriched by at least four fold over the negative control. The types of proteins identified at all three time points are summarized in Fig 3C and individual complexes and proteins are listed in Tables 1–6 and Table A in S1 Text. The most abundant types of proteins enriched on isolated viral DNA include those involved in RNA processing, transcription, chromatin remodeling, DNA repair, and DNA replication.

**Table 1:** HSV-1 proteins identified by iPOND and aniPOND.

HSV Proteins			iPOND (Mutant)								aniPOND (8hpi)					
			Experiment 1				Experiment 2				Experiment 1			Experiment 2		
			Ctrl	6hpi	8hpi	12hpi	Ctrl	6hpi	8hpi	12hpi	Ctrl	Mutant	KOS	Ctrl	Mutant	KOS
**Tegument**																
UL14	TEG3	P04291	0	0	4	4	0	2	5	2	4	15	3	0	7	4
UL21	TEG4	P10205	14	0	5	22	2	0	5	41	3	22	18	0	7	6
UL46	TEG1	P10230	10	6	6	30	6	7	8	39	0	86	28	3	101	115
UL47	TEG5	P10231	35	5	78	317	44	16	117	340	49	188	120	16	173	209
VP16	Transcription factor	P06492	4	11	16	85	21	6	52	108	9	86	81	13	127	158
ICP0	E3 ubiquitin-protein ligase	P08393	0	0	0	6	0	0	3	22	0	9	15	0	7	69
**Capsid**																
UL38	Triplex capsid protein VP19c	P32888	25	33	36	109	8	20	78	178	7	4	8	0	12	34
UL35	Capsid protein VP26	P10219					0	0	0	4	0	7	3	0	5	10
UL19	Major capsid protein VP5	P06491	151	302	284	588	137	239	376	615	66	93	129	18	219	262
UL6	Portal protein	P10190	5	11	21	28	0	7	9	21	0	93	4	4	180	94
**Capsid Assembly**																
UL37	Capsid assembly protein	P10221	0	0	0	2	0	0	0	11	0	0	21	0	0	30
UL26	Capsid scaffolding protein VP22a	P10210	0	6	7	40	0	6	16	34	0	0	5	3	6	15
**Packaging proteins**																
UL17		P10201	0	0	9	18	0	9	22	44	0	9	12	0	16	25
UL32		P10216	0	0	0	13	0	0	2	19	0	0	4	0	12	22
UL25		P10209	0	0	13	42	4	0	17	55	0	0	3	0	2	0
**Other viral proteins**																
UL2	Uracil glycosylase	P10210									0	0	16	0	0	47
UL3	Nuclear phosphoprotein	P10221	0	0	14	21	2	0	9	46	3	68	12	2	26	26
UL50	Deoxyuridine 5'-triphosphate nucleotidohydrolase	P10234	0	2	2	2	2	18	15	19	0	7	78	2	2	116
UL13	Serine/threonine-protein kinase	P04290									0	8	0	0	11	4
UL31	Virion egress protein	P10215	2	0	12	32	0	0	6	35	0	4	7	0	5	24

Experiments, conditions, and complexes are indicated in bold. Columns 1, 2, and 3 include protein name, description, and accession number. Values indicate spectral counts determined by mass spectrometry. Identified viral replication, transcription, and RNA processing factors are listed in Tables 2, 3, and 6, respectively.

**Table 2:** Replication factors identified by iPOND and aniPOND.

Replication Factors			iPOND-MS (Replication forks)				iPOND (mutant)								aniPOND (8hpi)					
			A		B	C	Expt 1				Expt 2				Expt 1			Expt 2		
DNA primase			ESCs	NIH3T3	293T	293T	Ctrl	6hpi	8hpi	12hpi	Ctrl	6hpi	8hpi	12hpi	Ctrl	Mutant	KOS	Ctrl	Mutant	KOS
POLA1	DNA polymerase alpha catalytic subunit	P09884	+	+	+															
POLA2	DNA polymerase alpha subunit B	Q14181	+	+																
PRIM2	DNA primase large subunit	P49643	+	+	+															
**DNA polymerase**																				
POLD1	DNA polymerase delta catalytic subunit	P28340	+	+	+	+												0	2	0
POLD2	DNA polymerase delta subunit 2	P49005	+		+															
POLD3	DNA polymerase delta subunit 3	Q15054	+		+															
POLE	DNA polymerase epsilon catalytic subunit A	Q07864	+	+	+	+														
**Clamp loader complex**																				
RFC1	Replication factor C subunit 1	P35251	+	+	+	+	0	2	2	0					0	0	4	0	2	2
RFC2	Replication factor C subunit 2	P35250	+	+	+	+									0	3	5	0	6	6
RFC3	Replication factor C subunit 3	P40938	+	+	+	+									0	0	4	0	4	11
RFC4	Replication factor C subunit 4	P35249	+	+	+	+									0	4	6	0	7	8
RFC5	Replication factor C subunit 5	P40937	+	+	+	+									0	4	3	0	9	7
**Replisome associated proteins**																				
LIG1	DNA ligase 1	P18858	+	+	+	+														
RPA1	Replication protein A 70 kDa DNA-binding subunit	P27694	+	+	+		0	7	0	3	0	0	2	9	0	0	0	0	14	22
TOP1	DNA topoisomerase 1	P11387					0	58	65	69	9	68	67	115	16	96	176	0	299	357
TOP2a	DNA topoisomerase 2-alpha	P11388					0	5	19	41	0	21	29	37	0	31	35	0	81	129
TOP2b	DNA topoisomerase 2-beta	Q02880	+	+			0	46	64	107	9	70	83	106	3	104	98	0	165	317
PCNA	Proliferating cell nuclear antigen	P12004	+	+	+	+	0	20	18	4	0	27	11	6	7	90	63	7	148	124
**MCM complex (helicase)**																				
MCM2	DNA replication licensing factor MCM2	P49736	+	+	+						0	0	0	2						
MCM3	DNA replication licensing factor MCM3	P25205	+	+	+		0	4	5	20	0	0	4	4						
MCM4	DNA replication licensing factor MCM4	P33991	+		+															
MCM5	DNA replication licensing factor MCM5	P33992			+		0	4	4	7	0	0	2	6						
MCM6	DNA replication licensing factor MCM6	Q14566	+	+	+															
MCM7	DNA replication licensing factor MCM7	P33993	+	+	+		0	0	4	2										
**HSV replication machinery**																				
ICP8	Major DNA binding protein	P04296					107	627	395	866	84	395	467	791	87	389	362	42	494	527
UL30	Polymerase	P04293					31	178	207	309	26	170	253	320	65	185	408	23	364	683
UL5	Helicase/primase	P10236					5	59	67	91	2	34	42	71						
UL8	Helicase/primase	P10192					0	0	9	18	0	0	0	9						
UL52	Helicase/primase	P10236					0	42	25	54	0	31	44	60						
UL9	Oriigin binding protein	P10193					0	34	21	66	0	36	45	90	8	219	201	22	390	406
UL42	Processivity factor	P10226					81	298	230	492	72	237	307	519	79	357	286	92	483	619

Experiments, conditions, and complexes are indicated in bold. Columns 1, 2, and 3 include protein name, description, and accession number. Values indicate spectral counts determined by mass spectrometry. Cellular replication fork iPOND-MS data was reported previously as follows: A [28], B [26], C [23].

**Table 3:** Transcription factors identified by iPOND and aniPOND.

**Transcription Factors**			**iPOND (Mutant)**								**aniPOND (8hpi)**					
			Experiment 1				Experiment 2				Experiment 1			Experiment 2		
DNA-directed RNA polyermase II			Ctrl	6hpi	8hpi	12hpi	Ctrl	6hpi	8hpi	12hpi	Ctrl	Mutant	KOS	Ctrl	Mutant	KOS
POLR2A	DNA-directed RNA polymerase II subunit RPB1	P24928	0	33	36	7	0	95	49	48	0	54	10	0	111	74
POLR2B	DNA-directed RNA polymerase II subunit RPB2	P30876	0	20	40	4	0	97	61	51	0	31	3	0	87	50
POLR2C	DNA-directed RNA polymerase II subunit RPB3	P19387	0	0	2	0	0	5	0	2	0	2	2	0	6	10
POLR2D	DNA-directed RNA polymerase II subunit RPB4	O15514	0	0	2	0					0	2	0	0	2	0
POLR2E	DNA-directed RNA polymerases II subunit RPB5	P19388	0	3	7	2	0	3	0	2	0	4	3	0	6	7
POLR2F	DNA-directed RNA polymerases II subunit RPB6	P61218									0	2	0			
POLR2G	DNA-directed RNA polymerase II subunit RPB7	P62487	0	2	0	0					0	5	0	0	7	5
POLR2H	DNA-directed RNA polymerases II subunit RPB8	P52434												0	2	2
POLR2I	DNA-directed RNA polymerase II subunit RPB9	P36954												0	7	2
POLR2J	DNA-directed RNA polymerase II subunit RPB11-a	P52435					0	2	2	0						
POLR2L	DNA-directed RNA polymerases II subunit RPB10	P62875					0	2	0	0						
**Mediator of RNA polymerase II transcription**																
MED1	Mediator of RNA polymerase II transcription subunit 1	Q15648									0	6	8	0	52	32
MED4	Mediator of RNA polymerase II transcription subunit 4	Q9NPJ6									0	2	0	0	7	6
MED6	Mediator of RNA polymerase II transcription subunit 6	O75586									0	4	0	0	12	7
MED7	Mediator of RNA polymerase II transcription subunit 7	O43513												0	7	0
MED8	Mediator of RNA polymerase II transcription subunit 8	Q96G25												0	7	5
MED10	Mediator of RNA polymerase II transcription subunit 10	Q9BTT4												0	4	0
MED11	Mediator of RNA polymerase II transcription subunit 11	Q9P086												0	3	2
MED12	Mediator of RNA polymerase II transcription subunit 12	Q93074									0	14	0	0	82	46
MED13	Mediator of RNA polymerase II transcription subunit 13	Q9UHV7												0	6	0
MED13L	Mediator of RNA polymerase II transcription subunit 13-like	Q71F56												0	11	0
MED14	Mediator of RNA polymerase II transcription subunit 14	O60244									0	22	10	0	156	119
MED15	Mediator of RNA polymerase II transcription subunit 15	Q96RN5												0	15	3
MED16	Mediator of RNA polymerase II transcription subunit 16	Q9Y2X0									0	18	9	0	51	31
MED17	Mediator of RNA polymerase II transcription subunit 17	Q9NVC6									0	9	5	0	37	27
MED18	Mediator of RNA polymerase II transcription subunit 18	Q9BUE0									0	3	3	0	12	6
MED20	Mediator of RNA polymerase II transcription subunit 20	Q9H944									0	6	4	0	11	11
MED21	Mediator of RNA polymerase II transcription subunit 21	Q13503									0	2	0	0	4	2
MED22	Mediator of RNA polymerase II transcription subunit 22	Q15528												0	4	3
MED23	Mediator of RNA polymerase II transcription subunit 23	Q9ULK4									0	59	27	0	132	110
MED24	Mediator of RNA polymerase II transcription subunit 24	O75448									0	35	12	0	83	65
MED25	Mediator of RNA polymerase II transcription subunit 25	Q71SY5												0	16	16
MED27	Mediator of RNA polymerase II transcription subunit 27	Q6P2C8									0	4	2	0	16	17
MED28	Mediator of RNA polymerase II transcription subunit 28	Q9H204									0	2	2	0	3	4
MED29	Mediator of RNA polymerase II transcription subunit 29	Q9NX70									0	3	2	0	8	10
MED30	Mediator of RNA polymerase II transcription subunit 30	Q96HR3									0	4	0	0	7	4
MED31	Mediator of RNA polymerase II transcription subunit 31	Q9Y3C7									0	7	6	0	13	8
CCNC	Cyclin-C	P24863									0	2	3	0	6	5
CDK8	Cyclin-dependent kinase 8	P49336												0	8	10
**Transcription initiation factor TFIID**																
TAF1	Transcription initiation factor TFIID subunit 1	P21675												0	5	12
TAF2	Transcription initiation factor TFIID subunit 2	Q6P1X5									0	9	0	0	18	25
TAF3	Transcription initiation factor TFIID subunit 3	Q5VWG9												0	0	3
TAF4	Transcription initiation factor TFIID subunit 4	O00268												0	5	0
TAF5	Transcription initiation factor TFIID subunit 5	Q15542									0	8	2	0	62	45
TAF6	Transcription initiation factor TFIID subunit 6	P49848									0	20	6	0	48	41
TAF9	Transcription initiation factor TFIID subunit 9	Q16594												0	8	10
TAF9B	Transcription initiation factor TFIID subunit 9B	Q9HBM6									0	8	3	0	6	0
TAF10	Transcription initiation factor TFIID subunit 10	Q12962									0	2	0	0	2	2
TAF12	Transcription initiation factor TFIID subunit 12	Q16514									0	4	0	0	2	3
TAF15	TATA-binding protein-associated factor 2N	Q92804														
TBP	TATA-box binding protein	P20226												0	12	17
**Transcription initiation factor TFIIH**																
XPB	TFIIH basal transcription factor complex helicase ERCC3	P19447									0	0	9	0	39	60
XPD	TFIIH basal transcription factor complex helicase ERCC2	P18074									0	0	4	0	36	45
CDK7	Cyclin-dependent kinase 7	P50613												0	15	19
MAT1	CDK-activating kinase assembly factor MAT1	P51948												0	8	9
CCNH	Cyclin-H	P51946									0	0	2	0	8	9
GTF2H1	General transcription factor 2H subunit 1	P32780												0	6	14
GTF2H2	General transcription factor 2H subunit 2	Q6P1K8									0	2	6	0	21	38
GTF2H3	General transcription factor 2H subunit 3	Q13889									0	0	3	0	13	13
GTF2H4	General transcription factor 2H subunit 4	Q92759									0	0	5	0	17	36
**Viral transcription regulators**																
VP16	Tegument protein VP16	P06492	4	11	16	85	21	6	52	108	9	86	81	13	127	158
ICP4	Major viral transcription factor	P08392	46	300	199	247	31	259	266	366	133	613	585	113	1146	1255
ICP22	Transcriptional regulator ICP22	P04485	8	60	18	46	6	51	69	74	0	26	17	0	29	44
**Other cellular transcription factors**																
TRIM28	Transcription intermediary factor 1-beta	Q13263	0	130	94	117	3	81	121	162	0	26	19	0	121	181
CTCF	Transcriptional repressor CTCF	P49711					0	2	6	24	0	26	12	0	41	54
TFII-I	General transcription factor II-I	P78347	0	69	61	73	0	35	49	73	0	3	0	0	21	10
SPT5	Transcription elongation factor SUPT5H (DSIF component)	O00267	0	25	7	6	0	33	20	27	0	15	2	0	37	21
SPT6	Transcription elongation factor SUPT6H	Q7KZ85	0	5	6	0	0	28	10	14	0	17	0	0	38	14
CSK21	Casein kinase II subunit alpha	P68400									0	7	3	0	9	12
CSK2B	Casein kinase II subunit beta	P67870									0	6	2	2	10	7
FUBP1	Far upstream element-binding protein 1	Q96AE4	2	25	32	47	7	32	31	39						
FUBP2	Far upstream element-binding protein 2	Q92945	9	41	22	46	6	27	32	38						
MATR3	Matrin-3	P43243	0	52	41	69	0	41	50	105	0	2	0	0	16	5
BCLF1	Bcl-2-associated transcription factor 1	Q9NYF8	0	0	14	15	0	12	13	29	10	23	14	16	54	47
CDC5L	Cell division cycle 5-like protein	Q99459	0	0	11	10	0	4	11	17	0	2	0	0	8	5
NONO	Non-POU domain-containing octamer-binding protein	Q15233	18	85	90	123	22	85	121	133	31	67	60	8	88	75
CN166	UPF0568 protein C14orf166	Q9Y224	0	9	5	10	0	2	3	14	0	2	6	0	7	16

Experiments, conditions, and complexes are indicated in bold. Columns 1, 2, and 3 include protein name, description, and accession number. Values indicate spectral counts determined by mass spectrometry.

**Table 4:** Chromatin remodeling factors and histones identified by iPOND and aniPOND.

Chromatin Remodeling Factors			iPOND (Mutant)								aniPOND (8hpi)					
			Experiment 1				Experiment 2				Experiment 1			Experiment 2		
FACT complex			Ctrl	6hpi	8hpi	12hpi	Ctrl	6hpi	8hpi	12hpi	Ctrl	Mutant	KOS	Ctrl	Mutant	KOS
SUPT16h	FACT complex subunit SPT16	Q9Y5B9	0	74	61	47	0	77	51	47	0	15	14	0	8	38
SSRP1	FACT complex subunit SSRP1	Q08945	0	47	42	37	0	35	29	49	0	5	18	0	9	41
**INO80 complex**																
INO80	DNA helicase (KIAA1259)	Q9ULG1									0	6	0	0	26	27
ARP5	Actin-related protein 5	Q9H9F9									0	3	0	0	3	3
ARP8	Actin-related protein 8	Q9H981												0	9	13
RUVBL1	RUVB-like 1 (TIP49A)	Q9Y265	8	46	40	47	0	13	21	24	0	93	39	0	104	112
RUVBL2	RUVB-like 2 (TIP49B)	Q9Y230	2	12	17	15	0	9	6	24	0	57	34	0	65	79
**NURD complex**																
CHD3	Chromodomain-helicase-DNA-binding protein 3	Q12873					0	0	0	9	0	0	9	0	23	41
CHD4	Chromodomain-helicase-DNA-binding protein 4	Q14839	0	28	22	42	0	36	35	61	0	57	77	0	81	124
HDAC1	Histone deacetylase 1	Q13547									0	32	15	0	25	24
HDAC2	Histone deacetylase 2	Q92769	0	0	0	6	0	12	8	22	0	9	0	0	79	79
RBBP4	Histone-binding protein RBBP4 (RbAp48)	Q09028	0	31	13	17	0	35	22	40	0	40	17	0	50	62
MTA1	Metastasis-associated protein MTA1	Q13330	0	0	0	11	0	3	5	3	0	21	10	0	45	28
MTA2	Metastasis-associated protein MTA2	O94776	0	11	19	23	0	5	11	19	0	27	22	0	61	67
**SWI/SNF complex**																
ARID1A	BAF250A (Swi1)	O14497	0	0	0	6	0	3	5	3	0	4	10	0	9	21
SMARCA4	BRG1 (Swi2/Snf2)	P51532	0	13	10	0	0	11	22	22	0	29	20	0	51	91
SMARCC1	BAF155 (Swi3)	Q92922	0	0	0	3	0	0	7	8	0	17	0	0	22	21
SMARCC2	BAF170 (Swi3)	Q8TAQ2	0	16	12	24	0	13	22	26	0	31	25	0	61	100
SMARCD2	Swp73	Q92925									0	3	3	0	3	12
ACTL6A	BAF53 (Swp61)	O96019	0	8	6	6	0	0	0	2	0	7	8	0	26	38
SMARCB1	BAF47 (Snf5)	Q12824									0	7	4	0	3	12
SMARCE1	BAF57	Q969G3	0	0	0	2	0	0	0	2	0	2	0	0	8	4
**COMPASS lysine methyltransferase complex**																
RBBP5	Retinoblastoma binding protein 5	Q15291									0	9	5	0	31	27
WDR82	WD repeat-containing protein 82	Q6UXN9	0	0	4	7	0	4	0	7				0	12	10
WDR5	WD repeat-containing protein 5	P61964	0	0	2	2					0	4	7	0	20	25
ASH2L		Q9UBL3									0	2	0	0	3	6
HCFC1	Host cell factor 1	P51610	0	0	4	0	0	0	0	6				0	6	11
MLL1	Histone-lysine N-methyltransferase 2A (Set1)	Q03164									0	6	0	0	2	2
**Chromatin/DNA modifying enzymes**																
DNMT1	DNA methyltransferase 1	P26358	0	6	3	2	0	11	14	4	0	0	4	0	0	7
PRMT1	Protein arginine methyltransferase 1 (ANM1)	Q9Y3Y2	2	20	15	30	2	21	15	29						
NAT10	N-acetyltransferase 10	Q9H0A0	0	0	4	10	0	4	8	12	0	5	5	0	11	3
MBB1A	Myb-binding protein 1A (HDAC activity)	Q9BQG0	0	0	4	7	0	7	15	22	0	73	21	0	13	10
KDM1A	Lysine-specific histone demethylase 1A (LSD1A)	O60341	0	3	0	4	0	0	2	0	0	21	13	0	62	64
APOBEC3C	DNA dC—> dU editing enzyme	Q9NRW3									0	11	7	0	6	8
**Chromatin associated and remodeling factors**																
HMGB1	High mobility group box 1	P09429	3	57	45	98	29	107	116	148				2	9	11
HMGB2	High mobility group box 2	P26583	0	12	0	6	0	28	26	46				0	3	6
CBX3	Chromobox homolog 3	Q13185	0	6	3	4	0	8	12	17	0	7	0	0	5	5
SMARCA5	hSNF2H	O60264	0	35	27	27	0	25	24	37	0	51	37	0	54	84
SMHD1	SMC hinge domain-containing protein 1	A6NHR9	0	41	42	23	0	43	31	42	0	28	37	0	106	111
DEK		P35659	0	2	2	13	3	20	14	21	0	11	13	0	57	42
**Histones**																
H1.2	Histone H1.2	|P16403	23	66	65	47	154	181	180	184	0	53	29	36	146	140
H1.4	Histone H1.4	P10412					115	142	142	156	0	26	30	11	153	135

Experiments, conditions, and complexes are indicated in bold. Columns 1, 2, and 3 include protein name, description, and accession number. Values indicate spectral counts determined by mass spectrometry.

**Table 5:** Cellular repair proteins identified by iPOND and aniPOND.

Repair Proteins			iPOND (Mutant)								aniPOND (8hpi)					
			Experiment 1				Experiment 2				Experiment 1			Experiment 2		
DSB recognition			Ctrl	6hpi	8hpi	12hpi	Ctrl	6hpi	8hpi	12hpi	Ctrl	Mutant	KOS	Ctrl	Mutant	KOS
KU80	XRCC5	P13010	6	106	79	104	2	88	96	120	0	32	23	0	38	51
KU70	XRCC6	P12956	0	79	64	103	0	42	52	85	0	8	10	0	24	46
**MRN complex—DSB repair**																
RAD50	Repair protein Rad50	Q92878	0	49	51	41	0	84	83	66	0	6	6	0	144	84
MRE11	Meiotic recombination 11 homolog	P49959	0	8	6	11	0	25	11	32				0	15	8
NBS1	Nibrin	O60934					0	2	0	0				0	2	2
**Mismatch repair**																
MSH2	MutS homolog 2	P43246	0	35	37	18	0	28	17	25	0	69	28	0	105	63
MSH3	MutS homolog 3	P20585									0	7	16	0	67	51
MSH6	MutS homolog 6	P52701	0	22	17	11	0	27	19	27	0	48	0	0	64	0
MLH1	MutL homolog 1	P40692	0	0	4	0	0	2	0	0						
**Base excision repair**																
PARP1	Poly [ADP-ribose] polymerase 1	P09874	0	40	50	70	0	35	42	60	6	108	108	0	296	265
XRCC1	X-ray repair cross-complementing protein 1	P18887	0	0	8	0	0	0	6	4	0	2	19	0	33	43
LIG3	DNA ligase 3	P49916	0	4	2	2	0	3	0	0	0	19	43	0	157	145
UL2	Viral uracil DNA glycosylase	Q1KMD3									0	0	16	0	0	47
APEX	DNA-(apurinic or apyrimidinic site) lyase	P27695	0	9	9	10	0	15	13	19	0	0	2	0	0	8
**Cohesin complex**																
SMC3	Structural maintenance of chromosomes protein 3	Q9UQE7	0	73	92	105	0	57	82	111	0	150	113	0	149	238
SMC1A	Structural maintenance of chromosomes protein 1A	Q14683	0	71	63	72	0	40	58	91	0	142	56	0	131	223
STAG1	SCC3A	Q8WVM7	0	0	0	2	0	0	9	10	0	63	42	0	44	67
STAG2	SCC3B	Q8N3U4					0	0	6	4	0	30	20	0	33	47
RAD21	SCC1	O60216					0	0	6	9	0	9	0	0	17	42
PDS5B	Cohesin complex interacting protein	Q9NTI5	0	3	0	12	0	22	27	55	0	42	50	0	107	158
**Other repair proteins**																
RECQL	ATP-dependent DNA helicase Q1	P46063	0	29	26	52	0	28	26	55	2	32	115	2	184	304
ATM	Ataxia telangiectasia mutated	Q13315	0	14	8	6	0	28	12	11	0	3	0	0	111	67
TRRAP	Transformation/transcription domain-associated protein	Q9Y4A5					0	0	0	2	0	34	18	0	141	113
DNA-PKcs	DNA-dependent protein kinase catalytic subunit	P78527	0	11	2	17	0	27	44	46	0	8	45	0	119	138

Experiments, conditions, and complexes are indicated in bold. Columns 1, 2, and 3 include protein name, description, and accession number. Values indicate spectral counts determined by mass spectrometry.

**Table 6:** RNA processing factors identified by iPOND and aniPOND.

RNA Processing Factors			iPOND (Mutant)								aniPOND (8hpi)					
			Experiment 1				Experiment 2				Experiment 1			Experiment 2		
TREX complex			Ctrl	6hpi	8hpi	12hpi	Ctrl	6hpi	8hpi	12hpi	Ctrl	Mutant	KOS	Ctrl	Mutant	KOS
THOC1	THO complex subunit 1	Q96FV9									0	6	6	0	14	25
THOC2	THO complex subunit 2	Q8NI27	0	18	3	19	0	0	5	12	0	39	18	0	88	66
THOC3	THO complex subunit 3	Q96J01									0	3	0	0	7	12
THOC4	THO complex subunit 4	Q86V81	0	0	0	2	13	21	20	23	16	8	10	51	65	80
THOC5	THO complex subunit 5	Q13769									0	2	0	0	21	17
THOC6	THO complex subunit 6	Q86W42									0	6	4	0	17	21
THOC7	THO complex subunit 7	Q6I9Y2									0	6	0	0	6	5
**Heterogeneous nuclear ribonucleoproteins**																
hnRNP AB	Heterogeneous nuclear ribonucleoprotein A/B	Q99729	0	43	23	46	8	31	20	41	5	28	4	0	22	25
hnRNP A0	Heterogeneous nuclear ribonucleoprotein A0	Q13151	0	0	7	14	7	15	11	30	5	13	0	0	16	21
hnRNP A1	Heterogeneous nuclear ribonucleoprotein A1	P09651	34	110	82	127	87	170	155	243	73	177	107	35	137	135
hnRNP A2/B1	Heterogeneous nuclear ribonucleoproteins A2/B1	P22626	30	88	69	124	93	183	174	248	74	235	100	33	180	199
hnRNP A3	Heterogeneous nuclear ribonucleoprotein A3	P51991	0	35	11	51	42	101	70	144	10	138	37	27	117	100
hnRNP C	Heterogeneous nuclear ribonucleoproteins C1/C2	P07910	40	134	123	176	38	116	114	176	53	174	71	55	135	127
hnRNP DL	Heterogeneous nuclear ribonucleoprotein D-like	O14979	0	8	4	24	7	15	30	37	0	5	4	4	11	10
hnRNP D	Heterogeneous nuclear ribonucleoprotein D0	Q14103	11	59	37	68	29	90	59	128	2	41	15	3	65	57
hnRNP F	Heterogeneous nuclear ribonucleoprotein F	P52597	2	5	23	6	4	41	41	63						
hnRNP H	Heterogeneous nuclear ribonucleoprotein H	P31943	29	91	74	106	52	140	116	173	27	92	55	0	98	79
hnRNP H2	Heterogeneous nuclear ribonucleoprotein H2	P55795	0	0	11	42	0	17	0	59				0	7	0
hnRNP H3	Heterogeneous nuclear ribonucleoprotein H3	P31942	0	0	17	16	7	34	18	28	10	34	10	3	22	16
hnRNP L	Heterogeneous nuclear ribonucleoprotein L	P14866	16	61	57	79	50	73	93	112	38	127	58	14	123	98
hnRNP M	Heterogeneous nuclear ribonucleoprotein M	P52272	32	162	121	155	25	128	137	165	20	150	34	2	80	55
hnRNP U	Heterogeneous nuclear ribonucleoprotein U	Q00839	48	161	114	195	105	183	235	267	28	105	71	16	127	121
hnRNP UL1	Heterogeneous nuclear ribonucleoprotein U-like protein 1	Q9BUJ2	0	17	8	19	0	19	16	17						
hnRNP UL2	Heterogeneous nuclear ribonucleoprotein U-like protein 2	Q1KMD3	2	21	18	29	4	31	29	53	4	36	10	5	63	60
**RNA helicases**																
DDX3X	ATP-dependent RNA helicase DDX3X	O00571	9	12	2	15	0	2	0	0	3	28	21	2	19	26
DDX5	Probable ATP-dependent RNA helicase DDX5	P17844	15	80	70	105	6	65	61	78	24	78	72	6	65	59
DDX17	Probable ATP-dependent RNA helicase DDX17	Q92841	9	80	74	92	27	87	94	98	0	18	11	0	24	22
DDX23	Probable ATP-dependent RNA helicase DDX23	Q9BUQ8	0	2	13	14	0	4	6	8	0	24	6	0	42	36
DDX42	ATP-dependent RNA helicase DDX42	Q86XP3	0	0	8	5	0	12	10	15						
DDX46	Probable ATP-dependent RNA helicase DDX46	Q7L014	0	4	7	16	0	0	10	14				0	20	13
DHX15	Putative splicing factor ATP-dependent RNA helicase	O43143	0	30	30	44					0	38	6	0	32	12
DHX9	ATP-dependent RNA helicase A	Q08211	31	106	103	143	31	96	103	123	44	153	87	7	99	87
**Splicing factors**																
ELAV1	ELAV-like protein 1	Q15717	0	9	11	16	6	17	19	20	7	49	8	5	38	17
KIAA1967	DBIRD complex subunit	Q8N163	0	0	10	4	0	2	6	11				0	0	2
LA	Lupus LA protein	P05455	0	3	6	8	0	6	3	21						
LEG1	Galectin 1	P09382	6	23	17	31	9	40	27	45	7	10	11	14	24	28
MAGOHB	Protein mago nashi homolog 2	Q96A72	0	0	0	2	0	6	5	6	3	15	4	0	10	4
PRP6	Pre-mRNA-processing factor 6	O94906	0	3	6	4	2	8	9	16	4	24	21	0	96	84
PRP8	Pre-mRNA-processing-splicing factor 8	Q6P2Q9	0	45	53	87	8	54	74	93	13	126	73	0	249	198
PRP19	Pre-mRNA-processing factor 19	Q9UMS4	0	26	34	30	0	9	26	43	0	16	11	3	46	36
PRP40A	Pre-mRNA-processing factor 40 homolog A	O75400	0	9	19	13	0	10	10	23	0	17	10	0	46	38
RALY	RNA-binding protein Raly	Q9UKM9	7	26	47	49	8	52	46	61	22	99	30	10	40	38
SF3A1	Splicing factor 3A subunit 1	Q15459	0	7	6	20	0	10	16	18	0	5	4	0	27	30
SF3A3	Splicing factor 3A subunit 3	Q12874	0	0	5	5	0	6	12	20	0	4	4	0	11	2
SF3B1	Splicing factor 3B subunit 1	O75533	0	24	35	50	0	23	25	40	9	39	14	0	46	30
SF3B2	Splicing factor 3B subunit 2	Q13435	0	16	23	10	0	13	18	20	0	3	0	5	20	20
SF3B3	Splicing factor 3B subunit 3	Q15393	0	29	23	31	2	35	33	41	7	60	19	21	124	105
SF3B14	Pre-mRNA branch site protein p14	Q9Y3B4	0	2	4	10	0	0	5	11	2	7	0	0	8	6
SNRPA1	U2 small nuclear ribonucleoprotein A'	P09661	0	7	9	10	0	10	7	14	3	4	8	4	13	10
SNRPB	Small nuclear ribonucleoprotein-associated proteins B and B'	P14678	0	18	2	18	23	36	19	47	2	24	4	4	36	30
SR140	U2 snRNP-associated SURP motif-containing protein	O15042	0	9	13	15	0	6	12	16	0	14	0	0	9	3
SRSF1	Serine/arginine-rich splicing factor 1	Q07955	0	14	8	18	10	41	28	55	15	53	27	14	65	47
SRSF2	Serine/arginine-rich splicing factor 2	Q01130	0	3	7	20	5	15	22	41	0	11	6	2	15	16
SRSF6	Serine/arginine-rich splicing factor 6	Q13247	4	8	12	22	9	34	29	40	28	73	45	13	62	62
SRSF7	Serine/arginine-rich splicing factor 7	Q16629	0	8	14	15	4	13	15	40	12	69	22	14	42	33
SRSF9	Serine/arginine-rich splicing factor 9	Q13242					0	9	8	20	4	27	9	6	26	19
SRSF10	Serine/arginine-rich splicing factor 10	O75494					0	0	2	2	0	5	0	0	6	5
TRA2A	Transformer-2 protein homolog alpha	Q13595									0	14	4	4	22	21
TRA2B	Transformer-2 protein homolog beta	P62995	0	4	2	16	3	18	17	22	13	59	18	9	50	45
U2AF1	Splicing factor U2AF 35 kDa subunit	Q01081	0	14	5	19	0	19	14	26	0	0	2	0	6	7
U2AF2	Splicing factor U2AF 65	P26368	0	0	5	11	0	0	7	13						
U520	U5 small nuclear ribonucleoprotein 200 kDa helicase	O75643	0	47	54	96	7	38	45	57	15	93	55	8	149	121
U5S1	116 kDa U5 small nuclear ribonucleoprotein component	Q15029	0	28	10	26	0	12	18	25	3	46	17	0	77	58
**Other RNA processing factors**																
ADAR	Double-stranded RNA-specific adenosine deaminase (DSRAD)	P55265	0	2	9	12	0	2	9	12	0	56	6	0	21	10
SRRT	Serrate RNA effector molecule homolog	Q9BXP5	0	14	16	23	0	12	21	33	5	30	16	0	48	43
IMDH2	Inosine-5'-monophosphate dehydrogenase 2	P12268									0	14	0	0	13	0
CPSF1	Cleavage and polyadenylation specificity factor subunit 1	Q10570									0	14	4	0	27	7
PABP1	Polyadenylate-binding protein 1	P11940	13	15	37	47	9	33	30	64	26	147	46	2	123	49
PABP4	Polyadenylate-binding protein 4	Q13310	0	0	3	4	0	5	10	18	0	44	8	0	30	0
NCBP1	Nuclear cap binding protein 1	Q09161	0	2	2	9	0	0	2	5	0	7	0	0	8	9
ILF3	Interleukin enhancer-binding factor 3	Q12906	4	33	26	48	48	106	92	134	21	111	52	24	133	90

Experiments, conditions, and complexes are indicated in bold. Columns 1, 2, and 3 include protein name, description, and accession number. Values indicate spectral counts determined by mass spectrometry.

Furthermore, proteins that mediate nuclear transport, components of the nuclear cytoskeleton, and HSV structural proteins were bound to viral genomes. Several trends are present in these data. First, the total number of proteins that were recovered increased with time of infection. This is consistent with increasing amounts of labeled DNA as replication proceeds, allowing for more sensitive detection of bound proteins. Second, there was a relative increase in proteins that function in post-transcriptional RNA processing, as well as viral structural proteins with time. The increase in viral structural proteins including tegument proteins, capsid assembly factors, portal protein (UL6), and capsid proteins reflects the packaging of nascent genomes at later times during infection (Table 1).

Comparison of proteins identified at each time point suggests that the individual proteins found on replicated/replicating viral genomes at 6, 8, and 12 hpi were relatively similar (Fig 3D). There are significant overlaps between the three different time points with most proteins identified at two or more of the times sampled. The biggest difference was seen at 12 hpi and this reflects the increase in structural proteins, as well as the larger number of proteins recovered by iPOND at this time point.

Comparative analysis of replication proteins found on replicated cellular and viral DNA reveals the specificity of isolation of proteins on viral DNA (Table 2). Cellular replication forks are enriched for cellular replication factors including components of cellular DNA polymerase, clamp loader complex, MCM complex, as well as other replisome-associated proteins such as topoisomerases and PCNA [23,26,28]. In contrast, in our studies viral DNA was enriched for all seven components of the viral replication machinery including: ICP8, UL30 (polymerase), UL5/UL8/UL52 (helicase/primase complex), UL9 (origin binding protein), and UL42 (processivity factor). The cellular counterparts to these viral proteins were not enriched on viral genomes. One exception to this is the cellular processivity factor, PCNA. This protein was enriched on viral genomes at all times tested with the highest levels at 6 hpi, decreasing with time. Furthermore, cellular topoisomerases TOP1, TOP2a, and TOP2b were abundant on viral genomes and likely play a role in virus replication or other process.

### aniPOND is an alternative method to purify viral genomes and associated proteins

Accelerated native iPOND (aniPOND) is a modified version of iPOND that is quicker and does not utilize crosslinking [33]. It involves native conditions during purification, while iPOND involves crosslinking and stringent wash conditions (Fig 1C and 1D). We therefore predicted that aniPOND would reveal a unique set of proteins involved in viral genome mechanics compared to iPOND because less direct interactors could be detected.

**Fig 4:**
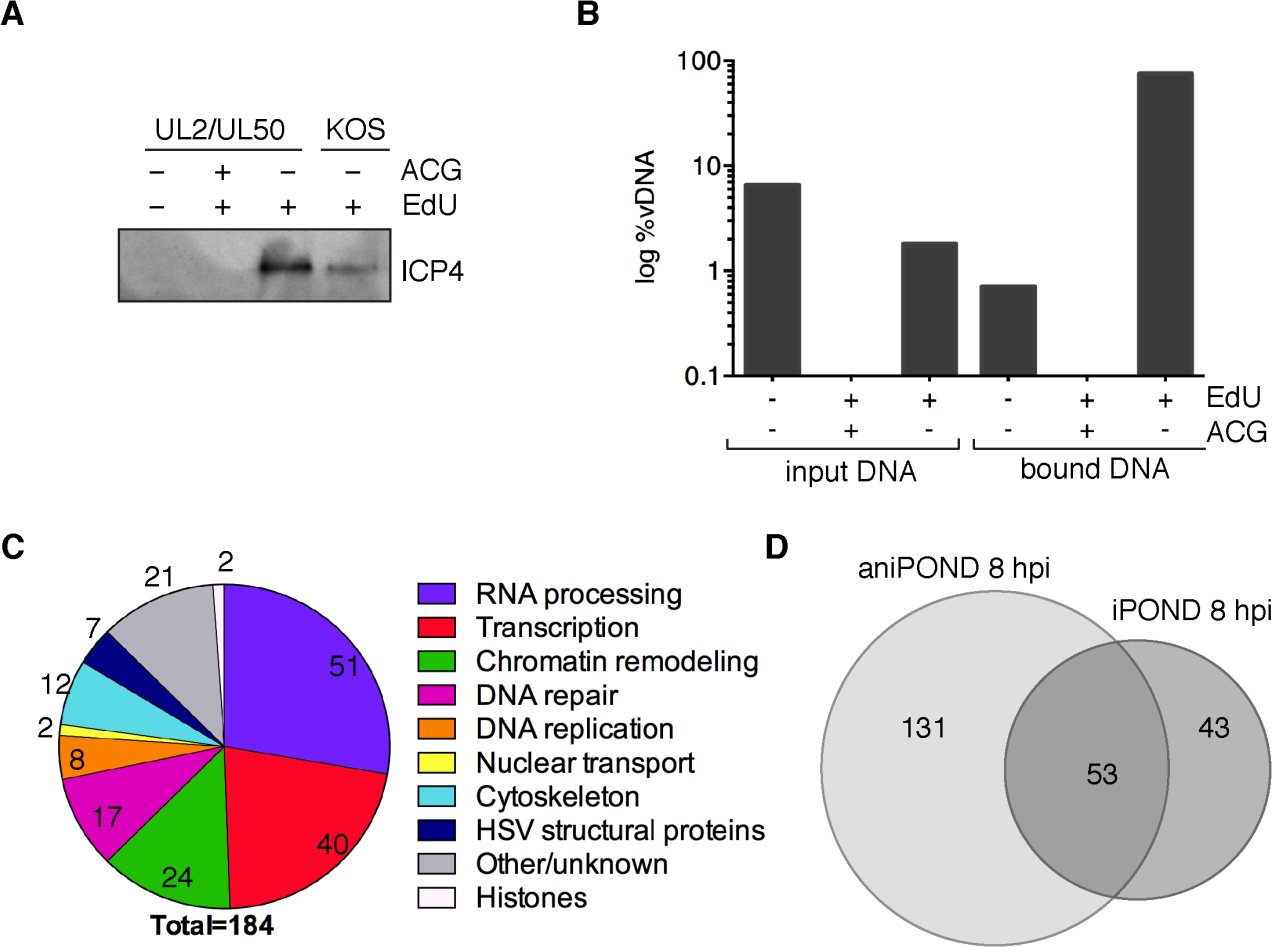
aniPOND detects viral and cellular proteins associated with replicated viral genomes. (A) ICP4 was detected in protein eluates from aniPOND experiments carried out on wild type KOS or UL2/UL50 mutant virus by western blotting. AniPOND was carried out on virus grown in the presence (+) or absence (-) of 2.5 μM EdU at 4–8 hpi and/or 33 μM acycloguanosine (ACG) throughout infection. (B) DNA eluted from streptavidin-coated beads during aniPOND experiments in (A) is viral. The amount of viral DNA present in cell lysates (input) and eluted from beads (bound) during aniPOND experiments was measured by qPCR of the viral TK gene. The ratio of viral DNA (vDNA) to total DNA was calculated and is presented in log %vDNA. Values for virus grown in the presence of ACG are too small to be displayed on this graph. These experiments were carried out with the UL2/UL50 mutant virus. (C) Pie charts summarize proteins that were identified by mass spectrometry of protein eluates from aniPOND carried out on the UL2/UL50 mutant grown in the presence of EdU at 4–8 hpi. Pie charts represent proteins that were identified with high confidence in independent duplicate experiments. Values indicate the number of proteins identified for each functional category. (D) Venn diagrams depict the overlap of proteins identified by iPOND and aniPOND carried out on the UL2/UL50 mutant 8 hpi.

To obtain a more comprehensive view of proteins bound to viral genomes, we carried out aniPOND on KOS and UL2/UL50 mutant viruses that were incubated in the presence of EdU from 4–8 hpi and harvested at 8 hpi. Proteins eluted from viral DNA during aniPOND were assayed for ICP4 by western blotting (Fig 4A). ICP4 was detected when the infection was carried out in the presence of EdU (lanes 3 and 4), but not in the absence of EdU (lane 1). Importantly, using aniPOND it was also possible to recover ICP4 associated with wild type genomes, however, greater amounts where recovered in the sample with the mutant virus extract. In this experiment, significantly less (~2%) sample was required to isolate a similar amount of ICP4 to that recovered with iPOND. Therefore aniPOND is more efficient for the recovery of labeled viral DNA and associated proteins than iPOND. This is in agreement with comparison of the purification of replisome-associated proteins by each method [33].

To verify the specificity of aniPOND for the purification of replicated viral genomes, we carried out aniPOND on cells infected with the UL2/UL50 mutant virus that was maintained in the presence of acycloguanosine (ACG, acyclovir), a potent inhibitor of viral DNA replication. In the absence of viral DNA replication, DNA was not recovered by aniPOND and ICP4 was not detected by western blotting (Fig 4A, lane 2). To further validate aniPOND for purification of viral genomes, we determined the relative amount of viral DNA/total DNA purified by this method. DNA eluted from beads during aniPOND experiments was almost 100% viral in nature (Fig 4B, bound DNA, +EdU-ACG). This is a significant enrichment compared to the percent viral DNA present in lysates for this condition (input DNA, <2%). Very little DNA was detected when aniPOND was carried out on virus grown in the absence of EdU (bound DNA,-EdU) or in the presence of ACG (+ACG), consistent with specific purification of replicated viral DNA by aniPOND.

To determine the identity of proteins that copurified with viral genomes, mass spectrometry was carried out on samples prepared by aniPOND of labeled UL2/UL50 mutant and wild type KOS genomes at 8 hpi. Two independent experiments were carried out for each virus, with an unlabeled virus negative control that was done on the same day with the same cells and reagents. Almost twice as many proteins were identified with high confidence by aniPOND compared to iPOND at 8 hpi (184 compared to 96). The types of proteins identified by aniPOND are summarized in Fig 4C and individual proteins are listed in Tables 1–6 and Table A in S1 Text. Proteins that copurified with viral genomes by aniPOND at 8 hpi share the same functional categories as proteins that were purified by iPOND. In fact, pie charts that summarize the findings from these two experiments show very similar trends (compare Figs 4C to 3C 8hpi).

Proteins that copurified with UL2/UL50 mutant genomes by iPOND and aniPOND at 8 hpi were compared (Fig 4D). Fifty-three proteins were identified by both methods, 131 by only aniPOND, and 43 by only iPOND. Differences in proteins identified by each method likely reflect differences in the nature of DNA-protein interactions. For example, the viral helicase/primase complex was identified by iPOND but not aniPOND (Table 2). Crosslinking during iPOND could capture transient DNA-protein interactions or interactions that are lost during purification, which may be the case for ATPases such as the helicase/primase complex. On the other hand, the mediator of RNA polymerase II complex, as well as components of general transcription factor TFIID and TFIIH were identified by aniPOND but not iPOND (Table 3). Members of these complexes may not be in direct contact with the viral genome or may bind in an orientation that is not conducive to crosslinking. We have shown previously that the mediator complex, TFIID, and TFIIH copurify with ICP4 from virus-infected cells [34,35]. Here we also confirmed that ICP4 coprecipitates with mediator and TFIID from virus infected resting MRC-5 cells, along with a subset of transcription and chromatin remodeling factors that copurify with viral DNA (Table B in S1 Text). Therefore, ICP4 may provide a means to target these complexes to viral DNA.

Comparison of proteins identified by aniPOND of mutant genomes and wild type genomes (Tables 1–6, Mutant vs. KOS) reveal similar trends and in almost all cases the same proteins were found to be associated with both genomes. In fact, the most obvious difference is that viral peptides for UL2 and UL50 gene products were not enriched on UL2/UL50 mutant genomes but were enriched on wild type genomes (Table 1). This provides validation for these mutants not expressing UL2 and UL50 gene products and supports the use of mutant genomes for the purification and identification of virus-associated proteins.

To provide support for the specificity of iPOND and aniPOND methods for the purification of bona fide viral genome associated proteins, we searched the Contaminant Repository for Affinity Purification (CRAPome) [36] for cellular proteins identified by these methods (Fig F in S1 Text). This web-based database includes 411 datasets of common contaminants present in negative controls for protein purification. Most proteins that were identified in this study were found in less than 20% of the negative control datasets, consistent with specific enrichment of viral genome associated proteins by these methods.

Taken together, aniPOND is an alternative method for the purification of virus-associated proteins and may be more useful in situations were few genomes are present (for example before DNA replication) or when genomes are not efficiently labeled with nucleoside analogs (for example wild type KOS). Furthermore, the combination of both methods reveals a comprehensive look at proteins associated with viral genomes.

### Cellular factors relocalize to viral replication compartments during HSV infection

**Fig 5:**
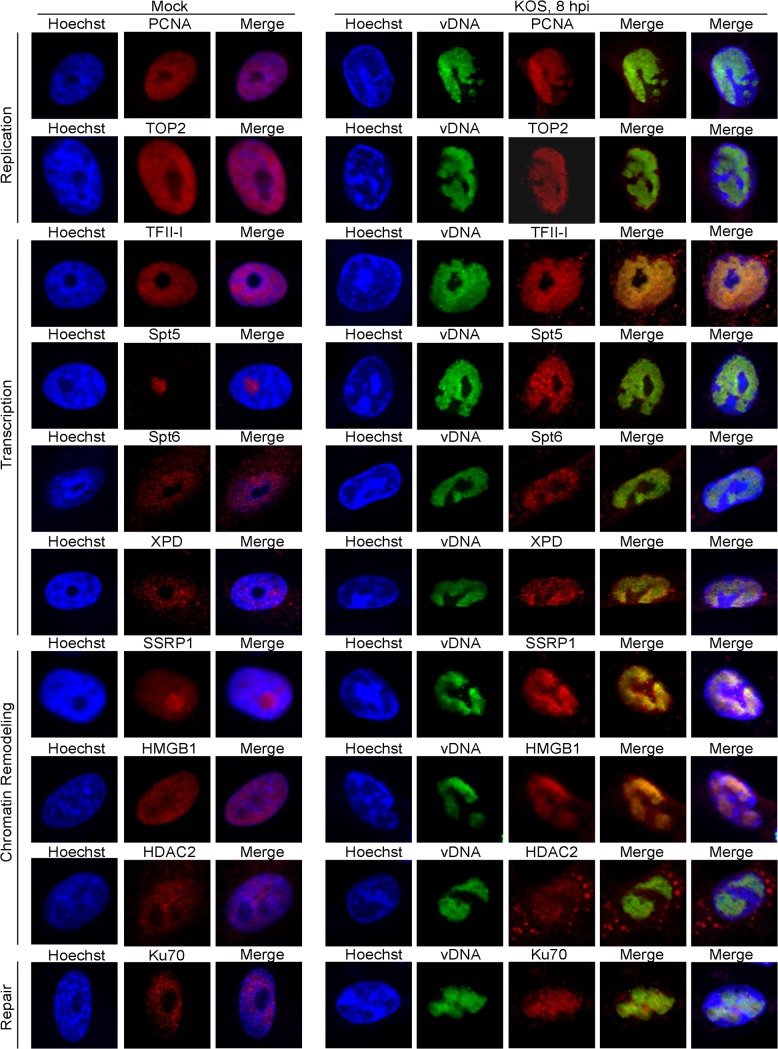
Proteins identified to interact with HSV-1 genomes by iPOND and aniPOND relocalize to viral replication compartments during lytic infection. Vero cells infected with wild type KOS were grown in the presence of 10 μM EdU at 4–8 hpi. Cellular DNA was visualized by Hoechst staining, viral replication compartments (vDNA) by click chemistry with EdU, and cellular proteins by immunofluorescence at 8 hpi (panels KOS, 8 hpi). Mock infected cells (panels Mock) display the normal distribution of cellular proteins in the nucleus in the absence of HSV infection. For KOS infected cells, the first merged panel displays colocalization of viral DNA with cellular proteins. The second merged panel shows the localization of viral DNA and cellular proteins with respect to cellular DNA.

To better visualize the reorganization of host nuclear factors to viral replication compartments during lytic infection with HSV, we used immunofluorescence to compare the distribution of cellular factors in the nucleus of mock-infected cells to cells infected with KOS for 8 hours (Fig 5). Ten cellular proteins that were identified by iPOND and/or aniPOND, including replication proteins PCNA and TOP2, transcription factors TFII-I, Spt5, Spt6, and XPD, chromatin remodeling factors SSRP1, HMGB1, and HDAC2, as well as the repair protein Ku70 were tested for colocalization with viral DNA. In all cases, the cellular proteins relocalized to viral replication compartments. Interestingly, these cellular factors were relocated from multiple locations within the nucleus. Taken together, it is clear that HSV infection induces gross reorganization of the host nucleus and compartmentalization of cellular factors that likely participate in multiple aspects of the virus life cycle.

### HSV genomes are deficient for histones during lytic infection

Micrococcal nuclease digestion assays indicate that packaged genomes are not associated with nucleosomes, only a small portion of incoming unreplicated genomes are associated with nucleosomes, and newly replicated genomes are not associated with nucleosomes [37–39]. However, ChIP mapping data indicate that histones are bound to many HSV promoters and genes, and often have marks of active chromatin [40,41]. The working model is that histones are present on viral genomes during early lytic infection, the distribution and density of histones on lytic genomes is significantly less than the host genome, and histones likely play a role in the regulation of viral gene expression. In contrast, latent genomes are associated with ordered chromatin similar to host cell DNA [42].

Many components of chromatin remodeling complexes were identified on replicated viral genomes by iPOND and aniPOND (Table 4). These include members of the FACT, INO80, NURD, and SWI/SNF chromatin remodeling complexes, as well as DNA and chromatin modifying enzymes. However, histones were not enriched on purified replicated genomes, with the exceptions of a few histone H1 variants, which were also abundant in negative controls (Table 4 and Fig F in S1 Text). Perhaps chromatin remodeling factors associate with viral DNA to facilitate the removal of histones or to keep histones from binding to newly replicated genomes.

**Fig 6:**
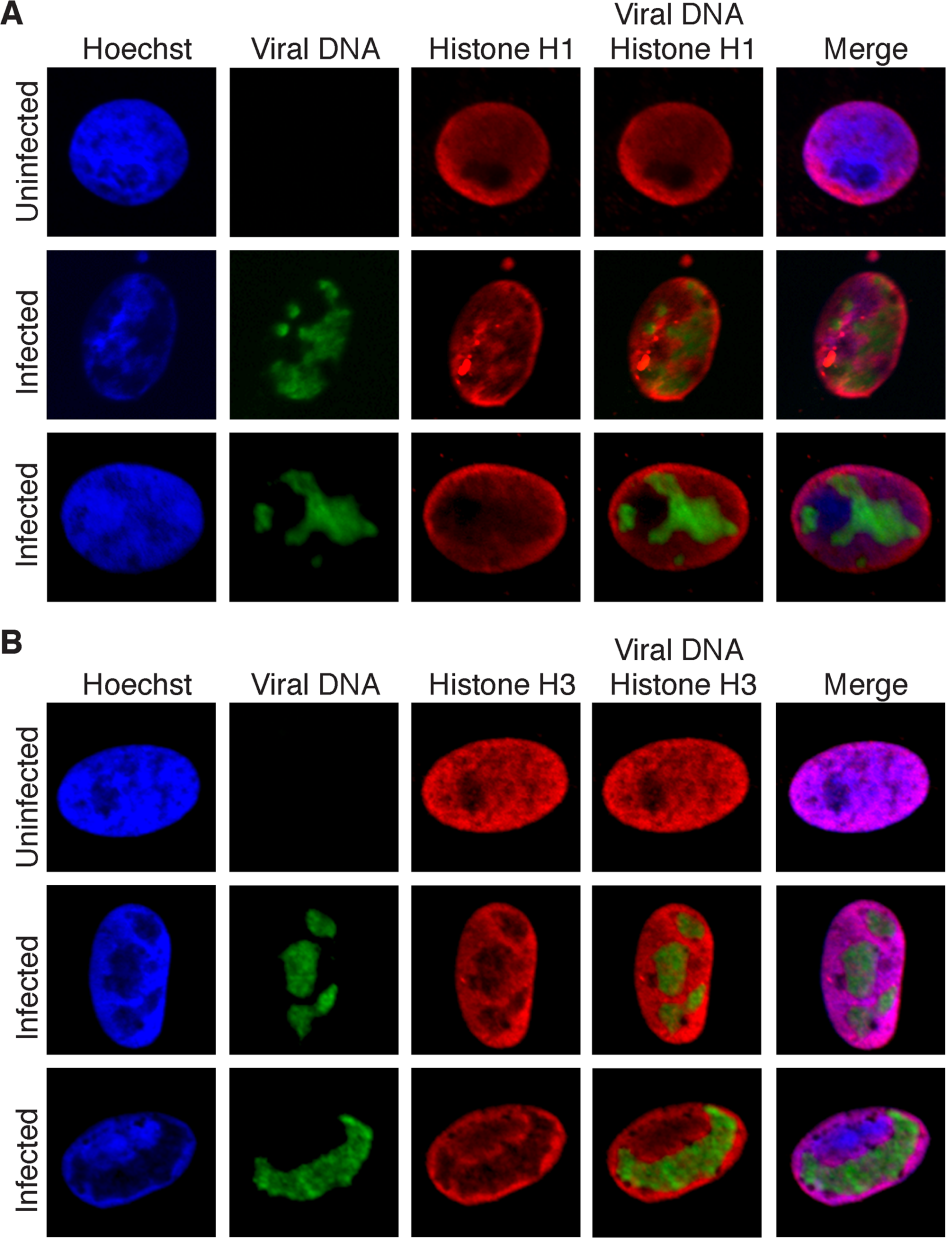
Histones H1 and H3 do not colocalize with replicated HSV genomes. (A) Infected Vero cells were maintained in the presence of EdU at 4–8 hpi. Cellular DNA was visualized by Hoechst staining, viral replication compartments by click chemistry with EdU, and histone H1 by immunofluorescence. Merged panels show the lack of colocalization of viral DNA with histone H1. Uninfected cells are shown as a control for normal histone distribution in the nucleus. (B) Imaging was carried out as in (A) except that immunofluorescence was carried out with antibodies specific for histone H3.

To provide support for the absence of histones on replicating genomes, colocalization of viral genomes with histones was assayed by fluorescence microscopy. EdU-labeled viral replication compartments were tagged with Alexa Fluor 488 and either histone H1 (all subtypes; Fig 6A) or H3 (6B) was labeled with specific antibodies for immunofluorescence. Less dense localization of both histones was observed with viral DNA relative to cellular DNA. This localization pattern greatly contrasts the pattern observed for proteins that were identified to associate with viral genomes (Fig 5). These data support iPOND and aniPOND results and confirm that histones are not enriched on viral genomes during DNA replication.

**Fig 7:**
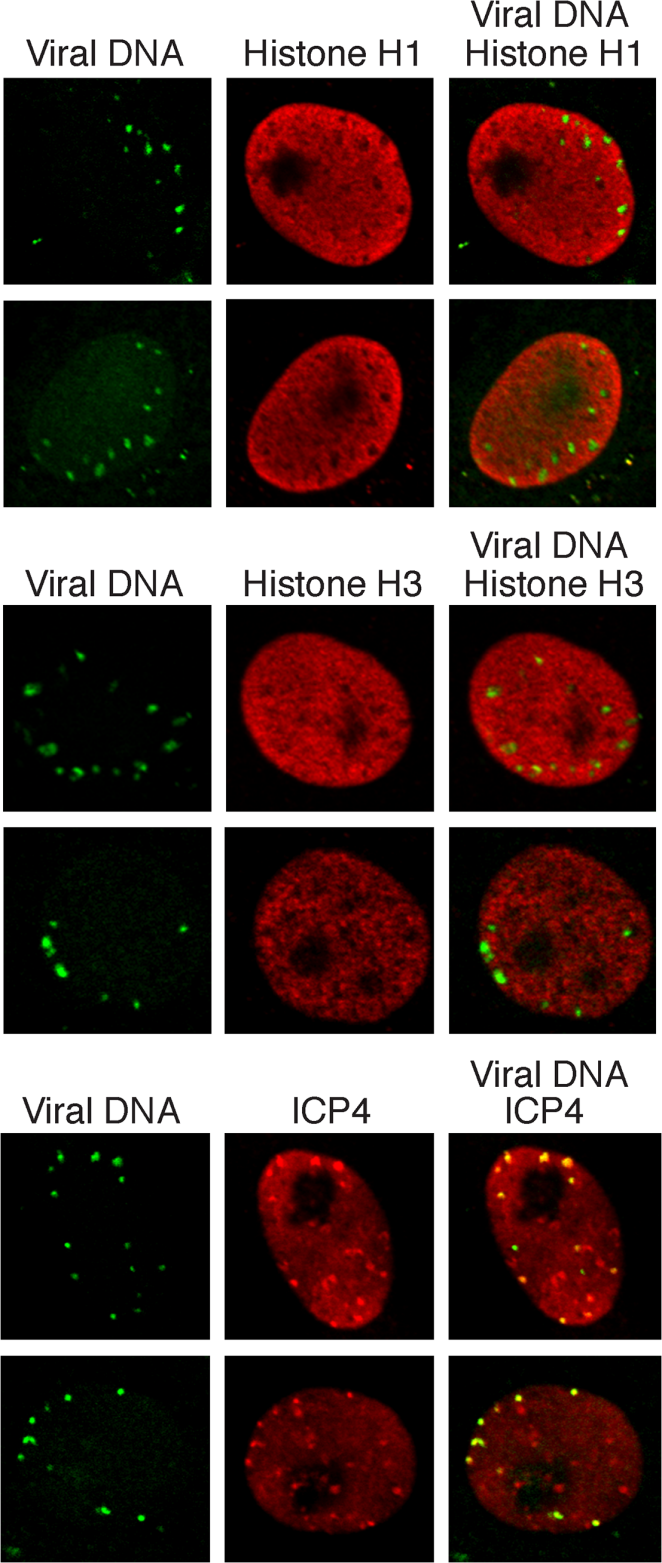
Histones H1 and H3 do not colocalize with incoming HSV genomes. Vero cells infected with KOS virus (prelabeled with 10 μM EdU) were assayed for colocalization with histones H1 and H3 at 2 hpi. Nuclei are shown with incoming viral genomes visualized by click chemistry with EdU, and histone H1, histone H3, or ICP4 by immunofluorescence. Merged panels show the lack of colocalization of viral DNA with histones and robust colocalization with ICP4.

To assay for the colocalizaiton of viral genomes with histones during early lytic infection, fluorescence imaging of prelabeled incoming viral genomes was carried out at 2 hpi (Fig 7). Histones H1 and H3 did not colocalize with incoming viral genomes, at least within limits of detection by immunofluorescence. This is in stark contrast to the pattern of ICP4 colocalization with incoming genomes. In conclusion, iPOND, aniPOND, and imaging data provide support for a deficiency of histones on viral genomes throughout lytic infection

## Discussion

In this study, we adapted procedures that have been used to label and purify cellular replication forks to label and purify replicating HSV-1 genomes. We have engineered mutant HSV strains that increase EdU incorporation into replicating viral genomes allowing for more sensitive imaging and purification of viral DNA. We are the first to label HSV DNA to track the fate of incoming viral genomes within an infected cell, demonstrating its colocalization with ICP4 expressed from those genomes. We have optimized the iPOND and aniPOND methods to study HSV genomes during different stages of the HSV life cycle by these methods. From these studies we have compiled a comprehensive list of proteins that are selectively recruited to HSV genomes during and after viral DNA replication. By imaging the relocalization of several of these factors to viral replication compartments during infection, we have demonstrated the extent to which host nuclei are largely reorganized during viral infection. Finally, we show that viral genomes isolated by iPOND and aniPOND have a relatively low abundance of histones, which is consistent with the lack of colocalization of genomes with histones H1 and H3. Data presented in this paper provide a comprehensive view of viral and cellular proteins that associate with replicating HSV genomes during productive infection and provide insight into how HSV manipulates host cell nuclear machineries for the expression, replication, and maintenance of its genome.

In this study, we identified >200 viral and cellular proteins that are associated with HSV genomes after the onset of DNA replication. The overall most abundant proteins found on the genome include the viral transcription factor ICP4 (Table 3), and the viral replication proteins UL29 (ICP8, major DNA binding protein), UL30 (viral DNA polymerase), and UL42 (processivity factor) (Table 2). The identified cellular proteins function in host cell nuclear processes including DNA replication, repair, chromatin remodeling, transcription, RNA processing, and nuclear transport (Figs 3C and 4C).

### Viral DNA replication

In addition to UL29, UL30, and UL42, four other viral replication factors, UL9 (origin binding protein) and UL5/UL8/UL52 (helicase primase complex) were enriched on viral genomes. In contrast to iPOND studies of cellular replication forks [23,26,28], most cellular DNA replication proteins did not copurify with viral genomes. However, the cellular processivity factor PCNA and topoisomerases TOP1, TOP2a, and TOP2b were reproducibly enriched on replicating viral genomes. iPOND data indicate that the levels of PCNA on viral genomes is higher at 6 hpi than at 8 and 12 hpi, suggesting that PCNA may play a role in early phases of viral DNA replication. Topoisomerases are important for relaxing supercoiled DNA as a consequence of helicase unwinding during replication and transcription [43], and likely carryout this same function on viral DNA. PCNA (Fig 5)[44] and Top2 (Fig 5) redistribute to viral replication compartments during viral DNA replication, however a direct role in HSV replication has yet to be demonstrated. Currently, there is not a good system to study origin-primed viral DNA replication *in vitro* [20]. It is possible that cellular PCNA or topoisomerases are the missing players in these reconstitution assays.

### The DNA damage response, repair, and recombination

We also identified several components involved in double strand break (DSB) recognition and repair associated with replicating viral genomes in our assays. These include Ku70 and Ku80, the Mre11/Rad50/Nbs1 (MRN) complex, ataxia telangiectasia mutated (ATM), and the catalytic subunit of DNA dependent protein kinase (DNA-PKcs) (Table 5). Ku70 (Fig 5) and Ku80 [45] colocalize with viral replication compartments. However, Ku70 expression is inhibitory for viral DNA replication [45]. Perhaps, these proteins participate in a cellular antiviral response in attempt to control virus multiplication.

The MRN complex, ATM, and activation of the DNA damage response are beneficial for HSV genome replication [46–48]. The MRN complex and ATM are recruited to viral replication compartments and ATM is activated through autophosphorylation to trigger the DNA damage response and cell cycle arrest through multiple pathways. In this way, the cell recognizes the viral genome as DNA damage. However, downstream binding of cellular proteins that mediate repair through nonhomologous end joining (NHEJ) and homologous recombination (HR) pathways are inhibited by the actions of the viral E3 ubiquitin ligase, ICP0 [49–51]. ICP0 targets downstream factors in these double strand break repair pathways for degradation, including DNA-PKcs, RNF8, and RNF168. Consistent with these data, we did not identify RNF8 or RNF168 to be recruited to viral DNA in our assays. Purification of DNA-PKcs is not inconsistent with these observations because only 50% of DNA-PKcs is degraded by ICP0 and this is likely cell type specific [45]. These data support a scenario whereby viral genomes trigger the DNA damage response and cell cycle arrest to create an environment that is conducive to viral DNA replication. ICP0 may inhibit the actions of cellular HR and NHEJ pathways for the repair of virus ends, as well as nicks and gaps that occur during viral DNA replication. It is possible that HSV-1 instead uses its own machinery for recombination and repair during DNA replication, mediated by the actions of ICP8 and UL12 (alkaline nuclease) [52,53]. In fact, UL12 has been shown to interact with components of the MRN complex and may therefore act with the MRN complex to carryout virus specific recombination [54].

The structural maintenance of chromosomes (SMC) family of ATPases function to stabilize and organize chromosomes during mitosis [55]. Of these complex members, SMC1 and SMC3, which make up the core of the cohesion complex, reproducibly copurify with replicating viral genomes. The cohesion complex is essential for sister chromatid cohesion during mitosis, but also plays a role in transcription and DNA repair by recombination [56]. Cohesin complex proteins SMC3 and Rad21 have previously been shown to associate with Epstein-Barr virus genomes [57,58]. Perhaps these proteins are involved in HSV gene expression or recombination during DNA replication. Mismatch repair [59] and base excision repair [60] pathways also function in maintaining HSV genomes, and specific factors involved in both of these types of repair were found to be associated with viral genomes in this study (Table 5).

### RNA processing

RNA processing factors involved in all steps in pre-mRNA processing including capping, splicing, polyadenylation, and export were abundant on viral genomes (Table 6). Interestingly, ICP27, an essential viral immediate early gene product that regulates all steps in viral RNA processing [61] was not readily detectable on genomes. However, the TREX complex was found in our studies, which has been shown to interact with ICP27 [62,63] and to be involved in the export of KSHV intronless mRNAs [64]. RNA helicases, which are involved in all aspects of RNA metabolism, as well as components of the nuclear transport machinery were also found associated with viral genomes. The abundant isolation of all of these RNA processing factors is most likely consistent with the high level accumulation of viral mRNA late after infection and the fact that transcription and RNA processing are coupled [65–67].

### Chromatin remodeling and transcription

Multiple components of several chromatin remodeling complexes were enriched on viral genomes including the FACT, INO80, NURD, and SWI/SNF complexes (Table 4). This is consistent with proteomic analysis of proteins bound to ICP4 extracted from virus infected cells, in which components of INO80, NURD, and SWI/SNF complexes were identified [34]. One of the FACT complex members, SPT16, was shown to copurify with ICP8 in the absence of DNAse treatment [45] and here we demonstrated the redistribution of the other FACT complex member SSRP1 to viral replication compartments (Fig 5). As discussed above, histones were not enriched on viral genomes, raising the possibility that these complexes maintain a nucleosome or histone free state, greatly facilitating processes such as replication and transcription on the genome. The FACT complex has been shown to disrupt nucleosome structure and allow DNA and RNA polymerases to access the DNA [68], the INO80 complex mediates nucleosome sliding [69], the NURD complex has both histone deacetylase and nucleosome remodeling functions [70], and high mobility group (HMG) proteins, which are also found on viral genomes, have been shown to increase accessibility of chromatin-bound DNA [71]. Furthermore, the INO80 and FACT complexes have also been implicated in cellular DNA damage repair by homologous recombination [72,73], and may therefore also play roles in mechanisms of viral DNA recombination. HMGB1 was previously shown to function as a coactivator for ICP4 mediated transcription *in vitro* [74], and may therefore function to mediate promoter specific activation of viral genes.

RNA polymerase II (polII) was abundant on isolated viral genomes (Table 3), with RPB1 and RPB2 being the most enriched subunits, most likely because they make direct contact with DNA during transcription [75]. The transcription elongation factors Spt5, Spt6 [76], and Trim28 [77] were also found associated with viral genomes and Spt5 and Spt6 were shown to relocalize to viral replication compartments (Fig 5). These are therefore likely candidates to regulate elongation during HSV transcription. TFII-I binds to initiator (inr) elements in cellular promoters [78] and therefore may play a role in the expression of late viral genes.

The viral transcriptional regulators VP16, ICP4, and ICP22 were found on viral genomes by both iPOND and aniPOND. ICP22 was previously found to associate with ICP4 and RNA polII in transcription complexes [79] and to mediate phosphorylation of polII [80]. VP16 is a tegument protein that activates transcription of immediate early viral genes [11]. ICP4 regulates expression from early and late HSV promoters and repression of immediate early promoters. It interacts with TFIID, TFIIH, and a specific form of the mediator complex that lacks Med26 and contains the kinase domain [34,35]. Here we show that viral genomes copurified with subunits of TFIID, TFIIH, and the same form of the mediator complex that copurified with ICP4 from Vero [34] and resting MRC-5 cells (Table B in S1 Text). This form of mediator possesses the kinase domain, but lacks med26, and thus may be involved in repression, possibly of immediate early promoters late after infection. The viral genes transcribed late after infection all possess relatively simple TATA box-containing promoters, yet are abundantly transcribed. The accumulated data support a model where ICP4 plays an integral role in recruiting most of the key polII transcription factors, such as TFIID, required for abundant late transcription.

This study has provided a comprehensive view of the viral and cellular proteins associated with replicating HSV genomes and provides new insight into cellular mechanisms that regulate HSV infection. The presence of cellular proteins involved in a variety of nuclear processes is consistent with the rapid and high level of accumulation of viral transcripts, replicated genomes, and progeny virions shortly after infection. This must be accompanied by the recombination and repair of replicating genomes. It is probable that the association and function of these factors is facilitated by the relative dearth of cellular chromatin, which may be a function of the recruitment of multiple chromatin remodeling complexes. In this model, ICP4 binding to the genome, may have multiple roles in recruiting chromatin remodeling complexes and key polII transcription complexes, although a direct role of ICP4 in chromatin organization has yet to be demonstrated. What remains to be studied is to what extent this state is determined prior to the onset of viral DNA replication.

## Materials and Methods

### Cells and viruses

Experiments were performed using MRC-5 (human embryonic lung) or Vero (African green monkey kidney) cells obtained from and propagated as recommended by ATCC. The viruses used in this study include the wild type HSV-1 strain, KOS, as well as UL2, UL50, and UL2/UL50 mutant viruses. Mutants were generated in bacterial artificial chromosomes (BACs) containing full-length, infectious KOS DNA [81] using two-step red-mediated recombination [82,83]. To generate the UL2 null virus, the cassette GGCTAGTTAACTAGCC, which contains a premature termination codon in all three reading frames, as well as an HpaI restriction site for validation, was inserted after the codon for cysteine 75 of the UL2 open reading frame. For the UL50 null virus, the codon for alanine 110 was replaced with this cassette. Mutant KOS-BAC constructs were transfected using Lipofectamine 2000 Transfection Reagent (Life Technologies) and propagated in Vero cells. Viral DNA was isolated from individual plaques [84] and screened for mutations by Southern blotting [85].

### Preparation of prelabeled viral genomes

To generate prelabeled virus stocks, 1x10^8^ Vero cells were infected with unlabeled KOS or UL2/UL50 at an MOI of 10 PFU/cell at 37°C for 1 hour. After rinsing with tris-buffered saline (TBS) to remove unadsorbed virus, media was replaced with Dulbecco’s Modified Eagle Medium (DMEM) containing 5% fetal bovine serum (FBS). Four hpi, EdU (Sigma-Aldrich) was added to the growth medium at the indicated concentration and incubated for an additional 34–36 hours. Monolayers were harvested, freeze-thawed three times at -80°C, sonicated, and clarified by low-speed centrifugation. Viral titers were determined by plaque assay on Vero cells.

### Click chemistry and immunofluorescence

A total of 2x10^5^ Vero cells were grown on glass coverslips in 12-well dishes. Infections were carried out at an MOI of 10 in 100 μl TBS for 1 hour at room temperature. After infection, inoculum was removed and cells were rinsed with 1 ml TBS prior to addition of 1 ml DMEM plus 5% FBS. Infections were carried out at 37°C for the indicated period of time, with or without the addition of EdU to the growth medium. Cells were fixed with 3.7% formaldehyde for 15 min, washed two times with phosphate-buffered saline (PBS), permeabilized with 0.5% Triton-X 100 for 20 min, and blocked with 3% bovine serum albumin (BSA) for 30 min. EdU-labeled DNA was conjugated to Alexa Fluor 488 azide using the Click-iT EdU imaging kit according to manufacturer’s protocol (Life Technologies). Cells were rinsed with PBS plus 3% BSA, then PBS, labeled with Hoechst 33342 (1:2000 dilution) for 30 min, washed two times with PBS, then incubated with primary antibody (mouse anti-ICP4: 58S, 1:500; mouse anti-histone H1: ab4269 (Abcam), 1:1000; rabbit anti-histone H3: ab1791 (Abcam), 1:1000; mouse anti-PCNA: sc-056 (Santa Cruz), 1:200; mouse anti-topoisomerase 2: NA14 (Calbiochem), 1:20; goat anti-TFII-I: sc-9943x (Santa Cruz), 1:200; rabbit anti-Spt5: A300-869A (Bethyl Laboratories), 1:200; rabbit anti-Spt6: ab32820 (Abcam), 1:200; rabbit anti-TFIIH p89 (XPD): sc-293 (Santa Cruz), 1:200; mouse anti-SSRP1: 10D1 (Biolegend), 1:200; rabbit anti-HMGB1: ab18256 (Abcam), 1:1000; rabbit anti-HDAC2: sc-7899 (Santa Cruz), 1:200; rabbit anti-Ku70: sc-9033 (Santa Cruz), 1:200) and Alexa Fluor 594-conjugated secondary antibodies (Santa Cruz, 1:500) as described previously [34]. Images were obtained using an Olympus Fluoview FV1000 confocal microscope.

### iPOND

iPOND was carried out as described previously [25] with the following modifications. For each condition, three 500 cm^2^ tissue culture dishes containing confluent monolayers of MRC-5 cells (~7x10^7^ cells/dish) were infected with UL2/UL50 double mutant virus at an MOI of 10 PFU/cell for one hour at room temperature. After adsorption, the inoculum was removed and cells were rinsed with TBS before addition of fresh DMEM plus 5% FBS. Cells were incubated at 37°C for the indicated period of time before addition of EdU at a final concentration of 2.5 μM. After incubation for an additional 2–4 hours, cells were fixed with 1% (wt/vol) formaldehyde in PBS for 15 min at room temperature, quenched with 125 mM glycine, and harvested by scraping. Cell permeabilization, click chemistry, cell lysis, sonication, and streptavidin capture were carried out as described except the samples were sonicated 6 times for 30 sec each at 7 watts using a Cole-Palmer ultrasonic processor with microtip. For each condition, samples from three plates were combined, and proteins were eluted from streptavidin-coated beads by boiling in 200 μl 2x SDS Laemmli sample buffer to reverse formaldehyde crosslinks.

### aniPOND

aniPOND was carried out as described previously [33] with the following modifications. For each condition, one 500 cm^2^ tissue culture dish containing a confluent monolayer of MRC-5 cells (~7x10^7^ cells) was infected with wild type KOS or UL2/UL50 double mutant virus at an MOI of 10 PFU/cell for one hour at room temperature. After adsorption, the inoculum was removed and cells were rinsed with TBS before addition of fresh DMEM plus 5% FBS. Cells were incubated at 37°C for four hours before the addition of EdU (Sigma-Aldrich) at a final concentration of 2.5 μM, followed by an additional four-hour incubation. To detach the monolayer and extract nuclei, 20 ml nuclear extraction buffer was added directly to each plate, incubated at 4°C for 15 min, and harvested by scraping. Cell washes, click chemistry, cell lysis, sonication, and streptavidin capture were carried out as described except for cell lysis cells were incubated for 30 min total in lysis buffer and sonicated 8 times for 30 sec each at 7 watts. Proteins were eluted from streptavidin-coated beads by boiling in 66 μl 2x SDS Laemmli sample buffer. For aniPOND experiment 2, two plates of MRC-5 cells were used for analysis and for protein elution, streptavidin-coated beads from both samples were combined and proteins were eluted in 66 μl 2x sample buffer to generate a 2x concentrated sample.

### Western blotting

SDS polyacrylamide gel electrophoresis and western blotting were carried out as described previously [86]. Proteins were transferred to polyvinylidine fluoride membranes (Amersham) for chemi-luminescent detection with ECL reagent (Amersham). For detection of ICP4, membranes were probed with the 58S polyclonal mouse antibody (1:5000 dilution).

### Mass spectrometry and data analysis

Mass spectrometry was carried out by MSBioworks. The entire sample was separated ~1.5cm on a 10% Bis-Tris Novex mini-gel (Invitrogen) using the MES buffer system. The gel was stained with coomassie and excised into ten equally sized segments. Gel segments were processed as described and analyzed by nano liquid chromatography with tandem mass spectrometry (LC/MS/MS) [87]. Data were searched using Mascot and Mascot DAT files were parsed into the Scaffold software for validation, filtering, and to create a nonredundant list per sample. Data were filtered at 1% protein and peptide level false discovery rates and requiring at least two unique peptides per protein. Proteins were considered most significantly enriched by iPOND or aniPOND based on the following criteria: 1) protein had at least 5 spectral counts (SpC) in the experimental sample, 2) protein was not detected in the control or was enriched over the control by at least four-fold based on dividing SpC values, and 3) was detected in duplicate experiments. Raw SpC data without normalization are presented in Tables 1–6 and Table A in S1 Text.

### DNA isolation and quantitative real-time PCR (qRT-PCR)

DNA was isolated from 1/20^th^ volume cell lysates or 1/10^th^ volume streptavidin-coated beads during iPOND and aniPOND experiments. For isolation of DNA from cell lysates, an equal volume of 2x SDS-bicarb solution (2% SDS, 0.2 M NaHCO_3_) was added to the sample and for isolation of bead-bound DNA, beads were resuspended in 1x SDS-bicarb solution. Samples were incubated at 65°C overnight, followed by extraction with phenol:chloroform:isoamyl alcohol (25:24:1) and chloroform:isoamyl alcohol (24:1). DNA was recovered using the MinElute PCR Purification kit (Qiagen). DNA concentrations were measure using a Quibit Fluorometer and the Qubit dsDNA HS Assay Kit (Life Technologies).

qRT-PCR was carried out as described previously [88]. The HSV-1 TK gene was amplified to estimate the amount of viral DNA in each sample. Primers used for amplification of the HSV TK gene were TkdsF1 (5´-ACCCGCTTAACAGCGTCAACA-3´) and TkdsR1 (5´-CCAAAGAGGTGCGGGAGTTT-3´). Standard curves were generated using purified KOS DNA.

## Supporting Information

S1 Text Includes Supporting Materials and Methods, Supplemental Figures A-F, and Supplemental Tables A and B. (DOCX)
