# Supplementary material for: Selective Recruitment of Nuclear Factors to Productively Replicating Herpes Simplex Virus Genomes
Source: PLoS Pathog. 2015 May 27;11(5):e1004939. doi: 10.1371/journal.ppat.1004939 (PMC4446364; doi:10.1371/journal.ppat.1004939)
Supplement: S1 Text — (DOCX) [file ppat.1004939.s001.docx]

**SUPPORTING INFORMATION**

**MATERIALS AND METHODS**

**Illumina sequencing**

DNA samples were prepared for deep sequencing using the TruSeq ChIP Sample Preparation Kit (Illumina) according to manufacturer’s protocol using 6.5 ng input DNA. The libraries were analyzed for length and concentration using an Agilent Bioanalyzer. Barcoded samples were mixed in equimolar concentration and sent to the Tufts University Core Facility for Illumina sequencing. FASTQ files were uploaded onto the Galaxy server ([galaxyproject.org](http://galaxyproject.org)) for analysis. The reads were mapped to the HSV-1 KOS reference genome (GenBank accession number JQ780693) using Bowtie. For alignments, the terminal repeats (TRL and TRS) of the HSV-1 genome were removed since they are redundant with the internal repeats. SAM tools were used to filter mapped reads and to convert files to BAM format. Genome coverage was visualized using the Integrative Genomics Viewer (Broad Institute).

**ICP4 purification**

Streptavidin affinity purification of TAP-wtICP4 was carried out as described previously [34] except that one 500 cm^2^ tissue culture dish containing a confluent monolayer of MRC-5 cells (~7x10^7^ cells) was infected with wild type KOS or TAP-wtICP4 at an MOI of 10 PFU/cell for 6 hours prior to harvesting cells, preparation of nuclear extracts, and affinity purification.

**SUPPORTING FIGURES AND TABLES**


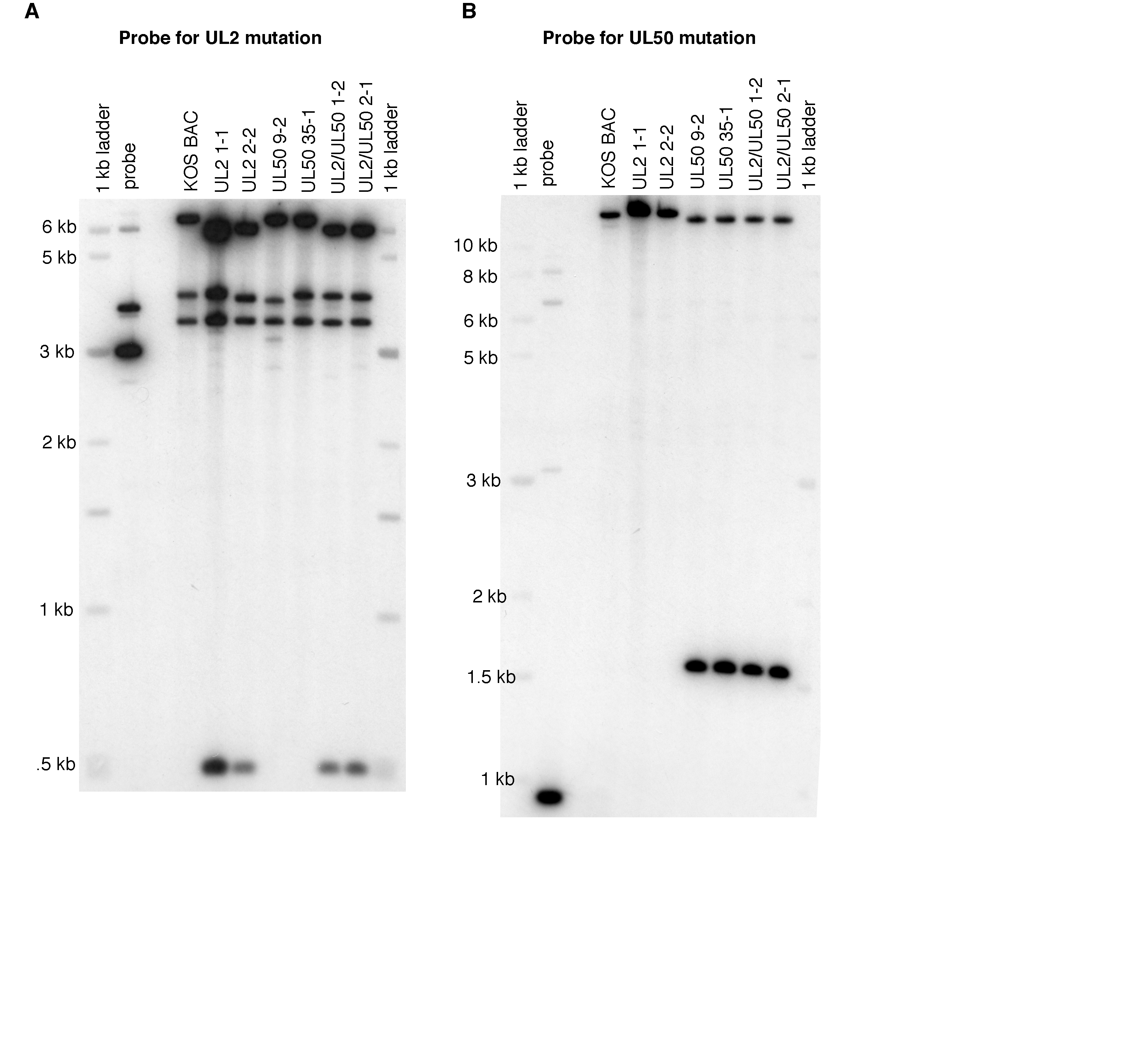


**Fig A. Engineering UL2 and UL50 mutant strains of HSV-1.**  (A/B) Southern blots to verify insertion of premature termination codons into the UL2 and UL50 open reading frames of the HSV-1 genome. Insertion of the stop codon cassette containing an HpaI restriction site was verified by Southern blot of HpaI digested viral DNA. (A) Insertion into the UL2 open reading frame results in cleavage of a 6.5 kbp fragment to 6.0 and 0.5 kbp. The probe for the UL2 mutation was generated by nick translation of the 3 kbp fragment that was gel purified from an HpaI/EcoRI digest of the EcoRI-C fragment of the HSV-1 genome. (B) Insertion into the UL50 open reading frame results in cleavage of a 15.2 kbp fragment to 13.6 and 1.6 kpb. The probe for the UL50 mutation was generated by nick translation of the 900 bp fragment that was gel purified from a BamHI digest of the EcoRI-I fragment of the HSV-1 genome. Strains UL2 2-2, UL50 35-1, and UL2/UL50 2-1 were used in this study.

**
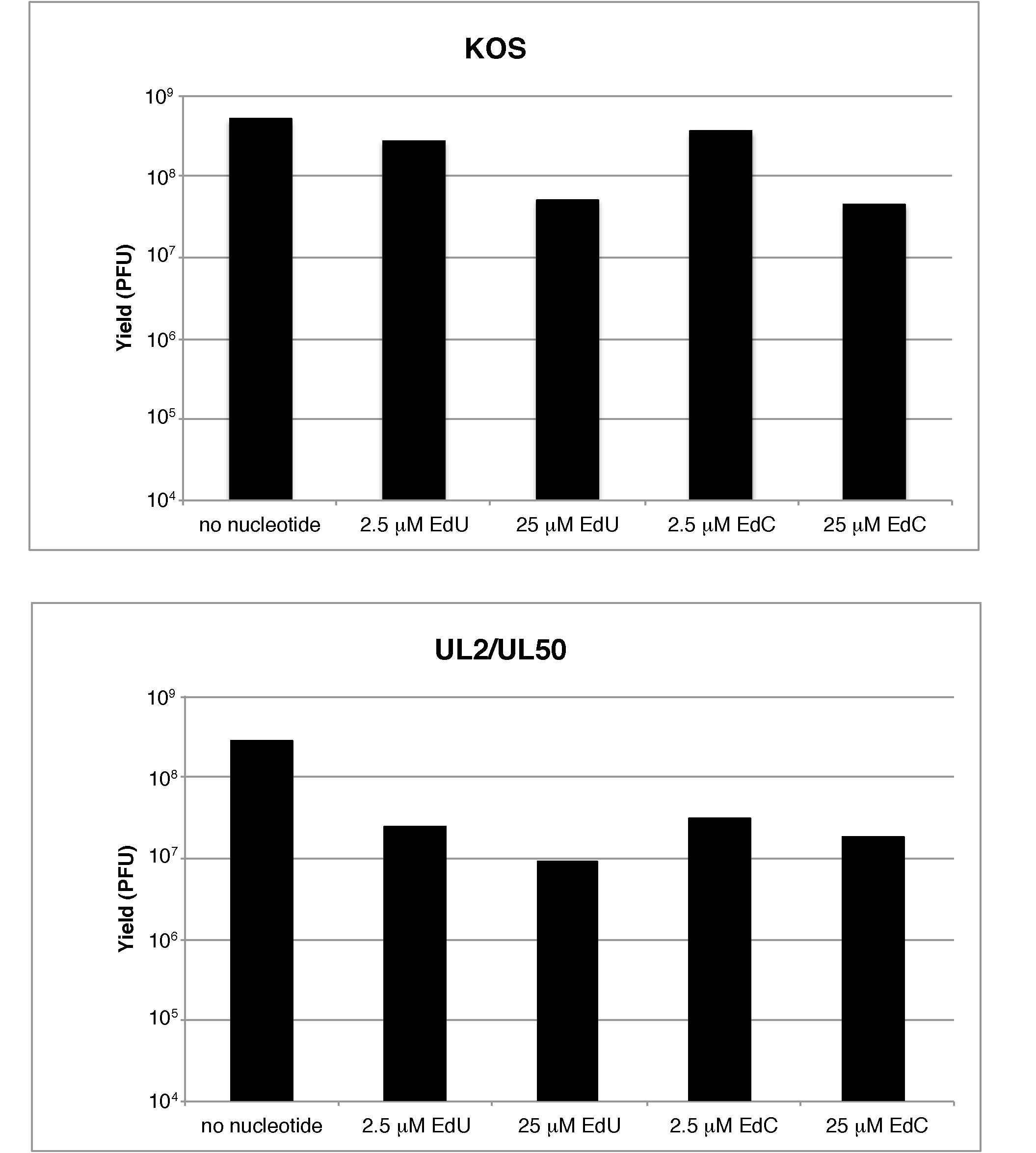
**

**Fig B. Effect of EdU and EdC incorporation on virus yield.** 1x10^6^ Vero cells were infected with wild type KOS or UL2/UL50 mutant virus at an MOI 10, followed by the addition of the indicated concentration of EdU or EdC to the growth medium at 4 hpi. Virus was harvested by freeze thaw method at 24 hpi and virus yield was determined by plaque assay in Vero cells.


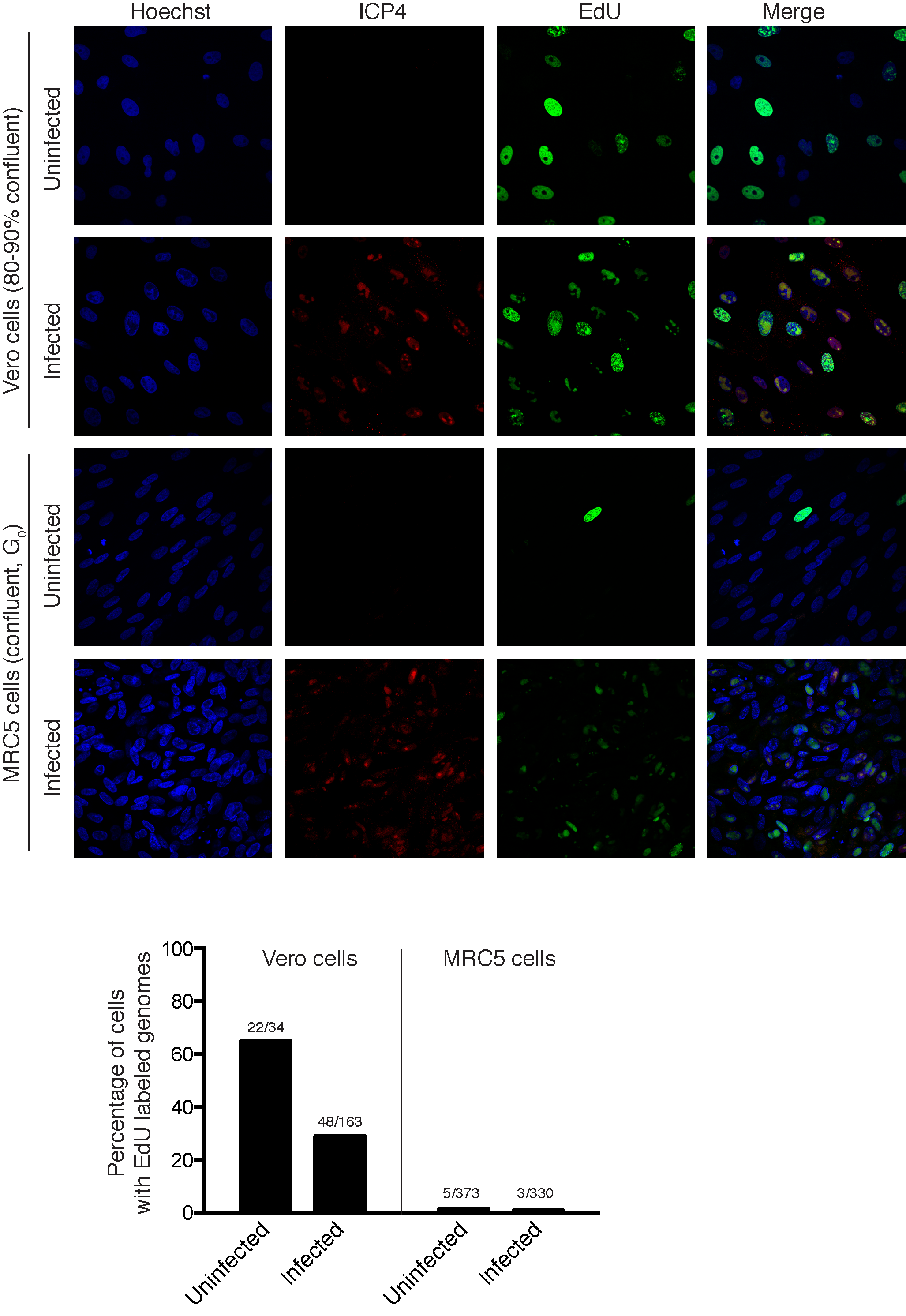


**Fig C. EdU is incorporated into replicating cellular DNA.** Uninfected or infected proliferating Vero cells (80-90% confluent) or resting MRC5 cells (confluent, G_0_) were assayed for genomic incorporation of EdU. EdU was added to the growth medium of UL2/UL50 mutant infected cells at 4-8 hpi or uninfected cells for 4 hours before fixation, click chemistry, and immunofluorescence. Cellular DNA was visualized by Hoechst staining, EdU by click chemistry with Alexa Fluor 488, and ICP4 by immunofluorescence. The percentage of cells with EdU labeled genomes was calculated by dividing the number of cells with nuclear EdU staining that colocalized with Hoechst stain over the total number of nuclei counted for each condition. Labeled viral DNA colocalizes with ICP4.


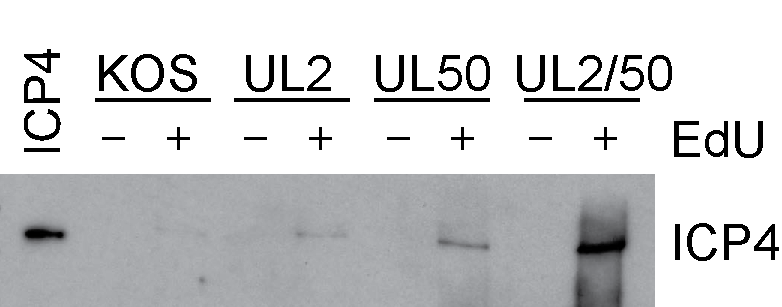


**Fig D. Relative protein yield from iPOND experiments.** iPOND was carried out on replicated wild type KOS, UL2 and UL50 single mutant, and UL2/UL50 double mutant genomes as described in the experimental procedures except that EdU was added to the growth medium at 4-8 hpi and iPOND was carried out 8 hpi. The negative control for each strain was iPOND carried out on unlabeled viral genomes harvested 8 hpi. Relative protein yield was determined by western blot for ICP4. The first lane contains purified ICP4.

**
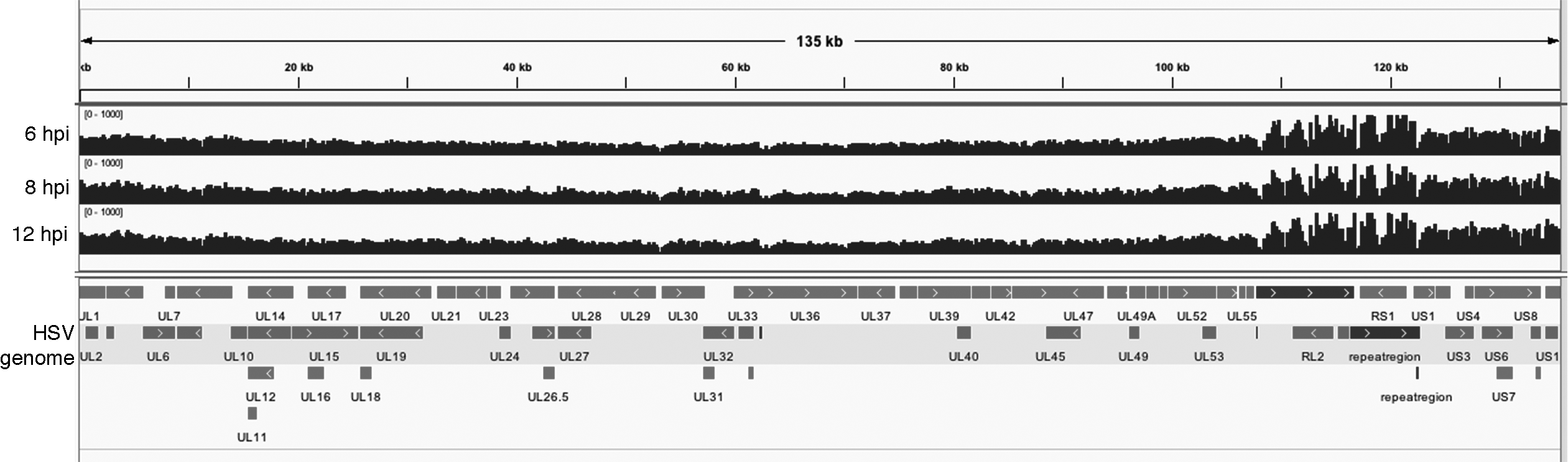
**

**Fig E. EdU is uniformly incorporated into HSV genomes during viral DNA replication.** Deep sequencing was performed on DNA eluted from streptavidin-coated beads during iPOND experiments in which labeled viral DNA was harvested at 6, 8, or 12 hpi. Sequence reads that mapped to the HSV-1 KOS genome were displayed using the Integrative Genomics Viewer (Broad Institute). Repeat regions of the HSV genome were deleted to simplify mapping and therefore peaks at these regions are twice as high.

**
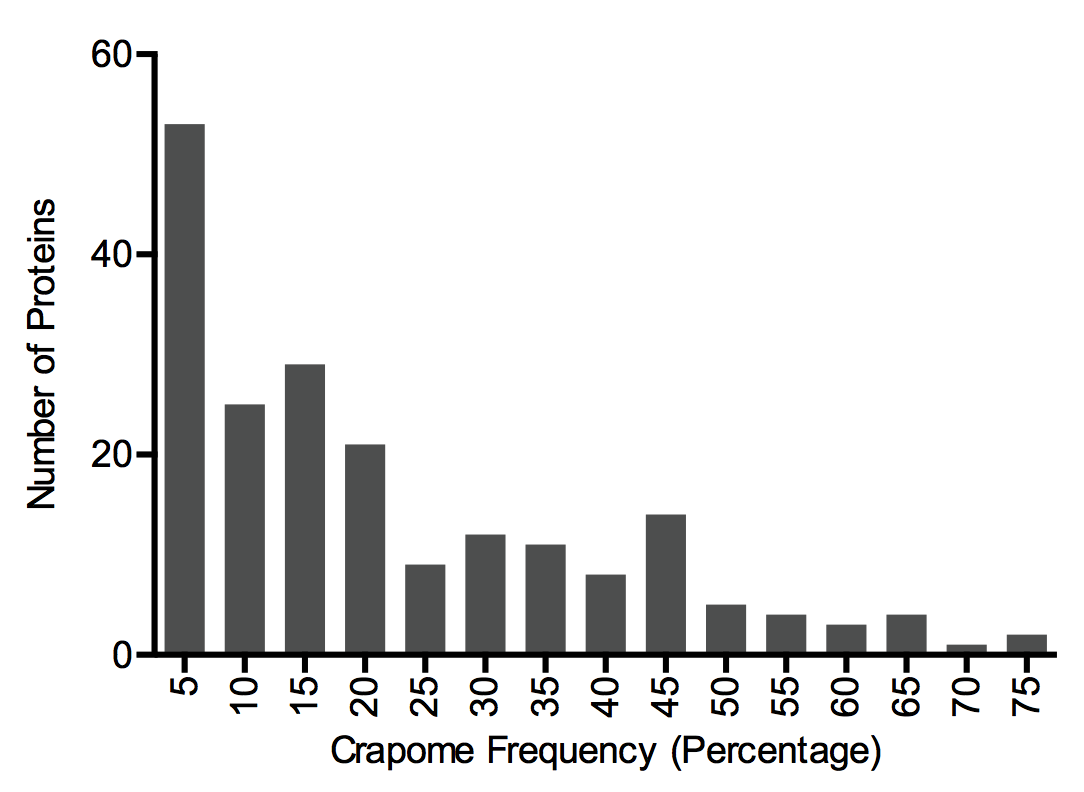
**

**Fig F. Proteins identified by iPOND and aniPOND are not enriched in the CRAPome database of common contaminants in affinity purification-mass spectrometry experiments.** Each protein identified to be associated with HSV genomes by iPOND- and/or aniPOND-mass spectrometry was searched against the CRAPome database [36] to determine the frequency by which that protein was identified in the 411 negative control datasets. The graph displays the distribution of the frequency by which individual proteins were identified in negative control datasets. Proteins that were identified in 5% or less of the control datasets were binned in group 5, 6-10% in group 10, 11-15% in group 15, and so on. Most proteins were identified in less than 20% of the negative control datasets, with the mean of 20% and the median of 15%. Proteins identified in over 50% of the negative control datasets include exclusively RNA helicases, hnRNP proteins, and histones. Viral proteins were not included in the CRAPome database search.

| **Cytoskeletal Proteins** | | | **iPOND (Mutant)** | | | | | | | | **aniPOND (8hpi)** | | | | | |
| --- | --- | --- | --- | --- | --- | --- | --- | --- | --- | --- | --- | --- | --- | --- | --- | --- |
|  |  |  | **Experiment 1** | | | | **Experiment 2** | | | | **Experiment 1** | | | **Experiment 2** | | |
|  | | | **Ctrl** | **6hpi** | **8hpi** | **12hpi** | **Ctrl** | **6hpi** | **8hpi** | **12hpi** | **Ctrl** | **Mutant** | **KOS** | **Ctrl** | **Mutant** | **KOS** |
| GSN | Gelsolin | P06396 | 23 | 40 | 11 | 27 | 14 | 18 | 15 | 31 | 0 | 5 | 7 | 0 | 39 | 45 |
| LMNB1 | Lamin-B1 | P20700 | 4 | 0 | 0 | 5 | 0 | 0 | 0 | 9 | 40 | 201 | 92 | 22 | 129 | 130 |
| LMNB2 | Lamin-B2 | Q03252 | 26 | 22 | 11 | 34 | 9 | 0 | 5 | 9 | 50 | 199 | 94 | 38 | 196 | 177 |
| LIMA1 | LIM domain and actin-binding protein 1 | Q9UHB6 |  |  |  |  |  |  |  |  | 0 | 11 | 0 | 0 | 11 | 2 |
| NUMA1 | Nuclear mitotic apparatus protein 1 | Q14980 | 0 | 32 | 42 | 49 | 0 | 12 | 14 | 28 | 0 | 5 | 4 | 0 | 30 | 35 |
| MYO1B | Unconventional myosin-Ib | O43795 | 11 | 0 | 0 | 3 | 0 | 2 | 0 | 0 | 0 | 11 | 2 | 0 | 19 | 10 |
| MYO1C | Unconventional myosin-Ic | O00159 | 32 | 23 | 5 | 12 | 8 | 11 | 4 | 2 | 4 | 53 | 19 | 2 | 107 | 108 |
| COF1 | Cofilin-1 | P23528 | 11 | 51 | 20 | 36 | 14 | 56 | 44 | 55 | 3 | 6 | 6 | 2 | 13 | 20 |
| LAP2A | Lamina-associated polypeptide 2, alpha | P42166 | 0 | 10 | 12 | 18 | 0 | 2 | 7 | 22 |  |  |  |  |  |  |
| LAP2B | Lamina-associated polypeptide 2, beta/gamma | P42167 |  |  |  |  |  |  |  |  | 0 | 10 | 8 | 0 | 3 | 0 |
| EMD | Emerin | P50402 |  |  |  |  |  |  |  |  | 2 | 12 | 4 | 2 | 10 | 11 |
| LEMD2 | LEM domain-containing protein 2 | Q8NC56 |  |  |  |  |  |  |  |  | 0 | 6 | 3 | 0 | 7 | 5 |

**Table A. Cytoskeletal proteins identified by iPOND and aniPOND.** Experiments, conditions, and complexes are indicated in bold. Columns 1, 2, and 3 include­ protein name, description, and accession number. Values indicate spectral counts determined by mass spectrometry.

|  | | | **KOS** | **ICP4-TAP** | **Complex** | **iPOND/**  **aniPOND** |
| --- | --- | --- | --- | --- | --- | --- |
| **RNA processing** | | |  |  |  |  |
| CPSF4 | Cleavage and polyadenylation specificity factor subunit 4 | O95639 | 0 | 2 | Cleavage and polyadenylation |  |
| CPSF5 | Cleavage and polyadenylation specificity factor subunit 5 | O43809 | 13 | 24 | Cleavage and polyadenylation |  |
| CPSF6 | Cleavage and polyadenylation specificity factor subunit 6 | Q16630 | 3 | 17 | Cleavage and polyadenylation |  |
| CPSF7 | Cleavage and polyadenylation specificity factor subunit 7 | Q8N684 | 6 | 12 | Cleavage and polyadenylation |  |
| FIP1L1 | Pre-mRNA 3'-end-processing factor FIP1 | Q6UN15 | 0 | 10 | Cleavage and polyadenylation |  |
| **Transcription** | | |  |  |  |  |
| ICP4 | Major viral transcription factor | P08392 | 9 | 1545 |  | Y |
| CSK21 | Casein kinase II subunit alpha | P68400 | 2 | 63 | Casein kinase II subunit | Y |
| CSK22 | Casein kinase II subunit alpha' | P19784 | 0 | 35 | Casein kinase II subunit | Y |
| CSK2B | Casein kinase II subunit beta | P67870 | 5 | 31 | Casein kinase II subunit |  |
| MED1 | Mediator of RNA polymerase II transcription subunit 1 | Q15648 | 0 | 8 | Mediator subunit | Y |
| MED4 | Mediator of RNA polymerase II transcription subunit 4 | Q9NPJ6 | 0 | 9 | Mediator subunit | Y |
| MED6 | Mediator of RNA polymerase II transcription subunit 6 | O75586 | 0 | 4 | Mediator subunit | Y |
| MED8 | Mediator of RNA polymerase II transcription subunit 8 | Q96G25 | 0 | 2 | Mediator subunit | Y |
| MED10 | Mediator of RNA polymerase II transcription subunit 10 | Q9BTT4 | 0 | 6 | Mediator subunit | Y |
| MED12 | Mediator of RNA polymerase II transcription subunit 12 | Q93074 | 0 | 2 | Mediator subunit | Y |
| MED15 | Mediator of RNA polymerase II transcription subunit 15 | Q96RN5 | 0 | 9 | Mediator subunit | Y |
| MED17 | Mediator of RNA polymerase II transcription subunit 17 | Q9NVC6 | 0 | 7 | Mediator subunit | Y |
| MED18 | Mediator of RNA polymerase II transcription subunit 18 | Q9BUE0 | 0 | 7 | Mediator subunit | Y |
| MED19 | Mediator of RNA polymerase II transcription subunit 19 | A0JLT2 | 0 | 4 | Mediator subunit |  |
| MED20 | Mediator of RNA polymerase II transcription subunit 20 | Q9H944 | 0 | 15 | Mediator subunit | Y |
| MED22 | Mediator of RNA polymerase II transcription subunit 22 | Q15528 | 0 | 9 | Mediator subunit | Y |
| MED24 | Mediator of RNA polymerase II transcription subunit 24 | O75448 | 0 | 14 | Mediator subunit | Y |
| MED25 | Mediator of RNA polymerase II transcription subunit 25 | Q71SY5 | 0 | 8 | Mediator subunit | Y |
| MED27 | Mediator of RNA polymerase II transcription subunit 27 | Q6P2C8 | 0 | 6 | Mediator subunit | Y |
| MED28 | Mediator of RNA polymerase II transcription subunit 28 | Q9H204 | 0 | 2 | Mediator subunit | Y |
| MED29 | Mediator of RNA polymerase II transcription subunit 29 | Q9NX70 | 0 | 4 | Mediator subunit | Y |
| MED30 | Mediator of RNA polymerase II transcription subunit 30 | Q96HR3 | 0 | 5 | Mediator subunit | Y |
| MED31 | Mediator of RNA polymerase II transcription subunit 31 | Q9Y3C7 | 0 | 8 | Mediator subunit | Y |
| CCNC | Cyclin C | P24863 | 0 | 2 | Mediator subunit | Y |
| TAF1 | Transcription initiation factor TFIID subunit 1 | P21675 | 0 | 51 | TFIID subunit | Y |
| TAF2 | Transcription initiation factor TFIID subunit 2 | Q6P1X5 | 0 | 2 | TFIID subunit | Y |
| TAF3 | Transcription initiation factor TFIID subunit 3 | Q5VWG9 | 0 | 11 | TFIID subunit | Y |
| TAF4 | Transcription initiation factor TFIID subunit 4 | O00268 | 0 | 33 | TFIID subunit | Y |
| TAF5 | Transcription initiation factor TFIID subunit 5 | Q15542 | 0 | 34 | TFIID subunit | Y |
| TAF6 | Transcription initiation factor TFIID subunit 6 | P49848 | 0 | 52 | TFIID subunit | Y |
| TAF7 | Transcription initiation factor TFIID subunit 7 | Q15545 | 0 | 11 | TFIID subunit |  |
| TAF8 | Transcription initiation factor TFIID subunit 8 | Q7Z7C8 | 0 | 7 | TFIID subunit |  |
| TAF9 | Transcription initiation factor TFIID subunit 9 | Q16594 | 0 | 24 | TFIID subunit | Y |
| TAF9B | Transcription initiation factor TFIID subunit 9B | Q9HBM6 | 0 | 14 | TFIID subunit | Y |
| TAF10 | Transcription initiation factor TFIID subunit 10 | Q12962 | 0 | 5 | TFIID subunit | Y |
| TAF11 | Transcription initiation factor TFIID subunit 11 | Q15544 | 0 | 8 | TFIID subunit |  |
| TAF12 | Transcription initiation factor TFIID subunit 12 | Q16514 | 0 | 9 | TFIID subunit | Y |
| TAF13 | Transcription initiation factor TFIID subunit 13 | Q15543 | 0 | 4 | TFIID subunit |  |
| TBP | TATA-box-binding protein | P20226 | 0 | 9 | TFIID subunit | Y |
| **Chromatin remodeling** | | |  |  |  |  |
| RUVBL1 | RuvB-like 1 | Q9Y265 | 0 | 8 | Ino80 subunit | Y |
| RUVBL2 | RuvB-like 2 | Q9Y230 | 0 | 7 | Ino80 subunit | Y |

**Table B. Factors that copurify with ICP4-TAP from virus infected resting MRC-5 cells.** Experiments, conditions, and complexes are indicated in bold. Columns 1, 2, and 3 include­ protein name, description, and accession number. Values indicate spectral counts determined by mass spectrometry.
